# Insights into the antigenic advancement of influenza A(H3N2) viruses, 2011–2018

**DOI:** 10.1038/s41598-019-39276-1

**Published:** 2019-02-25

**Authors:** Patricia A. Jorquera, Vasiliy P. Mishin, Anton Chesnokov, Ha T. Nguyen, Brian Mann, Rebecca Garten, John Barnes, Erin Hodges, Juan De La Cruz, Xiyan Xu, Jackie Katz, David E. Wentworth, Larisa V. Gubareva

**Affiliations:** 1 Influenza Division, Centers for Disease Control and Prevention (CDC), 1600 Clifton Road, Atlanta, GA 30329 USA; 2 CNI Advantage, LLC. 17 Executive Park Dr NE, Atlanta, GA 30329 USA; 3 Battelle Memorial Institute, 2987 Clairmont Rd, Suite 450, Atlanta, GA 30329 USA

## Abstract

Influenza A(H3N2) viruses evade human immunity primarily by acquiring antigenic changes in the haemagglutinin (HA). HA receptor-binding features of contemporary A(H3N2) viruses hinder traditional antigenic characterization using haemagglutination inhibition and promote selection of HA mutants. Thus, alternative approaches are needed to reliably assess antigenic relatedness between circulating viruses and vaccines. We developed a high content imaging-based neutralization test (HINT) to reduce antigenic mischaracterization resulting from virus adaptation to cell culture. Ferret reference antisera were raised using clinical specimens containing viruses representing recent vaccine strains. Analysis of viruses circulating during 2011–2018 showed that gain of an N158-linked glycosylation in HA was a molecular determinant of antigenic distancing between A/Hong Kong/4801/2014-like (clade 3C.2a) and A/Texas/50/2012-like viruses (clade 3C.1), while multiple evolutionary HA F193S substitution were linked to antigenic distancing from A/Switzerland/97152963/2013-like (clade 3C.3a) and further antigenic distancing from A/Texas/50/2012-like viruses. Additionally, a few viruses carrying HA T135K and/or I192T showed reduced neutralization by A/Hong Kong/4801/2014-like antiserum. Notably, this technique elucidated the antigenic characteristics of clinical specimens, enabling direct characterization of viruses produced *in vivo*, and eliminating *in vitro* culture, which rapidly alters the genotype/phenotype. HINT is a valuable new antigenic analysis tool for vaccine strain selection.

## Introduction

Influenza A viruses of the H3N2 antigenic subtype are important respiratory pathogens causing annual outbreaks of human illness since their emergence as a pandemic virus in 1968. The rapid evolution and accumulation of changes in the major surface antigens, hemagglutinin (HA) and neuraminidase (NA) result in antigenic drift, which is driven by escape from host immune response. Substitutions in the HA which result in escape from neutralizing antibodies are the major driver of antigenic drift^1^. At any given time point, there are multiple closely related genetic clades of HA genes expressed by co-circulating A(H3N2) viruses^2–5^.

The emergence of antigenic drift variants necessitates updates in vaccine composition to ensure optimal antigenic characteristics. Year-around surveillance of influenza viruses that cause seasonal epidemics is conducted by the Global Influenza Surveillance and Response System (GISRS) coordinated by the World Health Organization (WHO)^6^. The GISRS laboratories collect and characterize circulating influenza viruses. Representative viruses are shared with the WHO Collaborating Centres (CCs) who perform comprehensive genetic and antigenic characterization, as well as prepare vaccine candidate viruses. WHO CCs present their data at the bi-annual vaccine selection consultation meeting where decisions are made regarding the need for updating one or more vaccine components. These decisions require scientific evidence of antigenic drift and depend on availability of suitable candidate vaccine viruses^7,8^.

The vaccine manufacturing process requires 4–6 months, thus the vaccine selection decisions need to be made well in advance^9^. The antigenic similarity (match) between the viruses used in the quadrivalent or trivalent vaccines and viruses circulating during the following season is important for optimal vaccine effectiveness^9^. In addition, most influenza vaccines are prepared in fertilized chicken eggs, requiring adaptations of human viruses to eggs which results in the selection of viruses with altered HA receptor binding properties that may also exhibit changes in their antigenic characteristics^10^. Forecasting the major antigenic groups of influenza viruses that are most likely to dominate in the next season and producing suitable egg-propagated vaccine viruses is a daunting task, and various degrees of antigenic divergence (mismatch) have occurred over the years. This was the case for the Northern Hemisphere (NH) 2014–15 influenza season. For the 2013–2014 Northern Hemisphere season, the recommendation for the vaccine component was a cell-propagated A/Victoria/361/2011-like virus (HA genetic clade 3C), i.e. A/Texas/50/2012 (clade 3C.1)^7^. A/Texas/50/2012 well represented the majority A(H3N2) viruses circulating during the 2013–14 season and viruses collected and characterized during September 2013 and January 2014. Therefore, in February of 2014, A/Texas/50/2012 was again selected as the vaccine component for the 2014–2015 NH season. During the 2014–15 NH season, viruses from the HA genetic clades 3C.3, 3C.3a, 3C.3b, 3C.2a, and 3C.2b were co-circulating. Antigenic analysis showed that viruses expressing HAs belonging to clades 3C.3 and 3C.3b were antigenically similar to A/Texas/50/2012, while those carrying HAs from clades 3C.3a and 3C.2a were antigenically distinct^11,12^. Clade 3C.2a became the predominant group in many countries, including the U.S., leading to a significant vaccine mismatch and reduced vaccine effectiveness^6,13–15^.

In recent years, substantial efforts have been made to strengthen U.S. national and global surveillance. The number of laboratories participating in surveillance has increased, and the timeliness and representativeness of specimens submitted for virological characterization has improved, providing a positive impact on the overall quality of data^16,17^. The broad implementation of next generation sequencing (NGS) methods for characterization of virus genomes in clinical specimens and isolates propagated *in vitro*, and the use of advanced methods for genetic analyses are examples of these recent efforts. Special attention has been paid to the development and application of new assays to better characterize circulating influenza viruses^17,18^.

Rapid antigenic characterization of influenza viruses is important for detecting the emergence and spread of antigenic drift variants in a timely-manner, and it is essential for vaccine virus selection. Historically, antigenic monitoring of circulating viruses has been done using the hemagglutination inhibition (HI) assay; a test based on the ability of influenza virus particles to bind host receptors on red blood cells (RBCs) causing agglutination and the capability of the animal antisera to specific strains to block this agglutination. Using ferret antisera raised against the vaccine virus or a panel of reference viruses, circulating viruses can be characterized as antigenically similar (vaccine-like) or antigenically different from a vaccine strain. However, recent changes in the receptor binding characteristics of seasonal A(H3N2) viruses have diminished their ability to agglutinate RBCs, and also reduced/inhibited isolation and propagation in fertilized hen eggs^18^. Therefore, a large proportion of circulating A(H3N2) viruses cannot be analysed using the HI assay, creating the need for alternative methods of characterization. WHO influenza collaborating centres are now using virus neutralisation tests, in addition to hemagglutination-inhibition methods, to antigenically characterize circulating viruses for vaccine virus decision making purposes^19^. These methods still require multi-cycle propagation of viruses in cell culture which may introduce unwanted genetic changes in the HA or NA that can confound accurate characterization. A single-cycle replication approach has been utilized in a microneutralisation assay optimised for serological analysis^20^. However, this method requires a high multiplicity of infection (MOI of 2 to 3) to achieve ~100% infected cells and to measure neutralisation by an ELISA readout^20^.

In this study, we detail the antigenic characterization of influenza A(H3N2) viruses using a new assay, High-content Imaging-based micro-Neutralization Test (HINT), which was optimized to conditions of a single cycle infection using a low MOI. In addition, reference ferret antisera were raised using human respiratory specimens containing viruses from three antigenically and genetically distinct groups from 2014–2015. Together, these features of the HINT minimize the impact of host-cell adaptation on antigenic characterization. Using HINT and genomics we were able to assess antigenic relatedness among A(H3N2) viruses circulating during 2011–2018 and confirm the role of particular amino acid substitutions in the HA played in immune escape. We also demonstrated that this assay can be used to directly characterize viruses in primary human specimens, highlighting the future potential of this assay to antigenically characterize authentic virus populations and without the need for virus propagation in cell culture. This new technique is a promising approach to expedite detection of antigenic drift variants among rapidly evolving influenza A(H3N2) viruses.

## Results

### Characterization of reference material using high content imaging-based micro-neutralization test (HINT)

**Figure 1:**
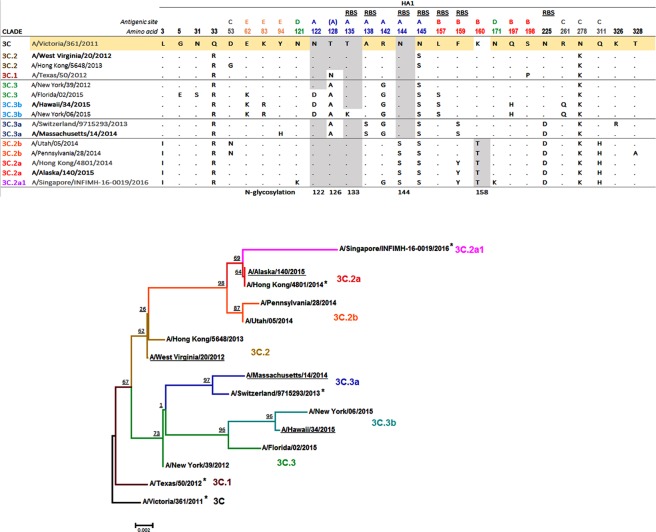
Representative A(H3N2) viruses utilized to generate HINT reference material. (**A**) HA1 sequences of human respiratory specimens selected to produce reference material (shown in bold) and cell-grown counterparts of viruses recommended as A(H3N2) vaccine strains during 2012–2017 (shown in grey). A/Victoria/361/2011 (M1: EPI ISL 101506), A/Texas/50/2012 (M1,C1: EPI ISL 101506), A/Switzerland/9715293/2013 (S1: EPI ISL 162149), A/Hong Kong/4801/2014 (S1: EPI ISL 165554); A/Singapore/INFIMH-16-0019/2016 (original: EPI ISL 225834) was recommended for the SH 2018 season. All viruses listed share sequons of N-glycosylation at residues 8, 22, 38, 45, 63, 165, 246, 246, 285 in HA1 and 154 in HA2. AA 128 belong to antigenic site B, however substitution T128N/A results in loss of glycosylation on N126 (antigenic site A). RBS: Receptor binding site. (**B**) Phylogenetic relationship among representative influenza A(H3N2) HA genes. Clinical specimens used in this study are underlined, and vaccines are marked with an asterisk. Branching patterns are color-coded by A(H3N2) HA genetic clade. Bootstraps per parent node and the scale bar (in nucleotide mutations per site) are indicated.

Human respiratory specimens containing viruses that were expected to be antigenically similar to one of three cell-propagated vaccine viruses A/Texas/50/2012 [TX/2012] (Clade 3C.1), A/Switzerland/9715293/2013 [SW/2013] (clade 3C.3a) or A/Hong Kong/4801/2014 [HK/2014] (clade 3C.2a) (Fig. 1) were used to generate reference antisera by inoculating ferrets (n = 4) with 10^3^ infectious units (IU). Respective reference virus stocks were generated by a short, 24 h period of culture in MDCK-SIAT1 (SIAT1) cells, as described in methods. HINT was carried out in SIAT1 cell cultures, due to their higher expression level of α-2,6-linked sialic acid receptors compared to regular MDCK cells, which makes them the preferred cell line to culture contemporary seasonal A(H3N2) viruses^21^. Briefly, viruses were mixed with serially diluted ferret antisera or diluent (no serum control), pre-incubated for one hour at room temperature, combined with a freshly prepared cell suspension and seeded onto 96-well plates. Notably, TPCK-treated trypsin was omitted from virus culture medium to prevent the spread of virus progeny to neighbouring cells (a single-cycle replication). Following a 24 h incubation at 37 °C, cells were fixed, cell nuclei were stained with Hoechst 33342 and virus-infected cells were detected by immunostaining against influenza NP. NP-positive (virus-infected cells) and NP-negative cells (not infected) were quantified using automated microscopy with a high-content imaging microplate reader. HINT titres were determined by calculating the reciprocal dilution of the antiserum needed to reduce the infected cell population (ICP) by 50% (IC_50_) compared to the control wells with no serum (100% infection) by curve fitting analysis.

Individual ferret antisera were repeatedly tested to determine homologous and heterologous HINT titres (Fig. S1). A single antiserum (α-serum) from each group that best discriminated between homologous and heterologous viruses was selected for subsequent experiments (Fig. S1, Table 1). For reference purposes, MDCK-propagated A/Texas/50/2012 (TX/2012) vaccine virus and its corresponding antiserum were obtained from International Reagent Resource (IRR, https://www.internationalreagentresource.org/) and included in the test. As anticipated, α-TX/2012 serum neutralized well the reference viruses from clade 3C.2, A/West Virginia/20/2012 (WV/20-3C.2), and clade 3C.3b, A/Hawaii/34/2014 (HI/34-3C.3b), characterizing these viruses as TX/2012-like (Table 1). The α-WV/20 and α-HI/34 reference sera showed very similar inhibition of WV/20 and HI/34 viruses, and exhibited 3- to 5-fold reduction in neutralization of TX/2012 (Table 1).

**Table 1:** Characterization of the reference ferret post-infection antisera.

Viruses	clade	Ferret antiserum, HINT titre (fold down)				
		pooled	F4	F5	F9	F13
		α-TX/2012	α-WV/20	α-HI/34	α-MA/14	α-AK/140
		3C.1	3C.2	3C.3b	3C.3a	3C.2a
A/TX/50/2012	3C.1	**5626 (1)**	3735 (5)	6908 (3)	1376 (1)	286 (25)
A/WV/20/2012	3C.2 1	6739 (0.3)	**20165 (1)**	25833 (1)	2388 (1)	1851 (4)
A/HI/34/2015	3C.3b	7386 (1)	12430 (2)	**21682 (1)**	1168 (1)	273 (26)
A/MA/14/2014	3C.3a	625 (9)	479 (42)	397 (55)	**1550 (1)**	236 (31)
A/AK/140/2015	3C.2a	695 (8)	713 (28)	547 (40)	434 (4)	**7224 (1)**

The α-TX/2012 serum showed 8- to 9-fold reduction in neutralizing ability against the reference viruses A/Massachusetts/14/2014 (MA/14-3C.3a), and A/Alaska/140/2015 (AK/140-3C.2a) (Table 1). These viruses belong to HA clades 3C.3a and 3C.2a, previously described as antigenically distinct from TX/2012^22–24^. Reference serum α-HI/34-3C.3b, showed ≥40-fold reduction in neutralization of MA/14-3C.3a and AK/140-3C.2a (Table 1). Notably, α-MA/14-3C.3a serum neutralized TX/2012, WV/20-3C.2 and HI/34-3C.3b as well as the homologous antigen, and showed a 4-fold reduction in neutralization of AK/140-3C.2a (Table 1). Furthermore, α-AK/140-3C.2a serum exhibited very low neutralization ability against TX/2012 (25-fold-reduction), HI/34-3C.3b (26-fold), and MA/14-3C.3a (31-fold), whereas, it showed only a 4-fold reduction in neutralization of WV/20-3C.2 (Table 1).

In summary, the reference antisera raised against WV/20-3C.2 and HI/34-3C.3b shared a neutralization profile similar to α-TX/2012, demonstrating viruses from clade 3C.1, 3C.2, and 3C.3b as antigenically similar and viruses from clades 3C.3a and 3C.2a as antigenically drifted. Therefore, only one serum, α-HI/34-3C.3b, was chosen to constitute part of the reference antisera panel (TX/2012-like antiserum). Sera raised against MA/14-3C.3a and AK/140-3C.2a were also included in the reference panel due to their ability to distinguish antigenic differences between 3C.3a and 3C.2a viruses. Due to the high level of amino acid sequence similarity (99.2%) between MA/14-3C.3a and SW/2013 HA1, and between AK/140-3C.2a and HK/2014 HA1 (100%) (Fig. 1A), these antisera are referred to as SW/2013-like and HK/2014-like in this study.

The reference panel of three antisera and respective viruses was used to assess reproducibility and variability of HINT (Fig. S2). Fifteen independent tests were conducted by two operators on different days using the same equipment. HINT results were analysed as neutralization titres and fold-differences in titres compared to homologues pairs (serum and virus). The coefficient of variation was much lower when data was converted to fold differences (Fig. S2). Thus, the results of the subsequent experiments are shown as fold difference in titres. Mean and standard deviation (SD) values of the fold difference in titres were determined for each reference serum and virus. A cut-off value of the mean plus five SD was chosen to set a high specificity. Thus, a virus was considered antigenically distinguishable when the ability of the clade-specific antiserum to neutralize that virus was reduced by >4-fold compared to the matching reference antigen.

### Antigenicity of A(H3N2) viruses representing distinct HA genetic groups, 2011–2018

**Figure 2:**
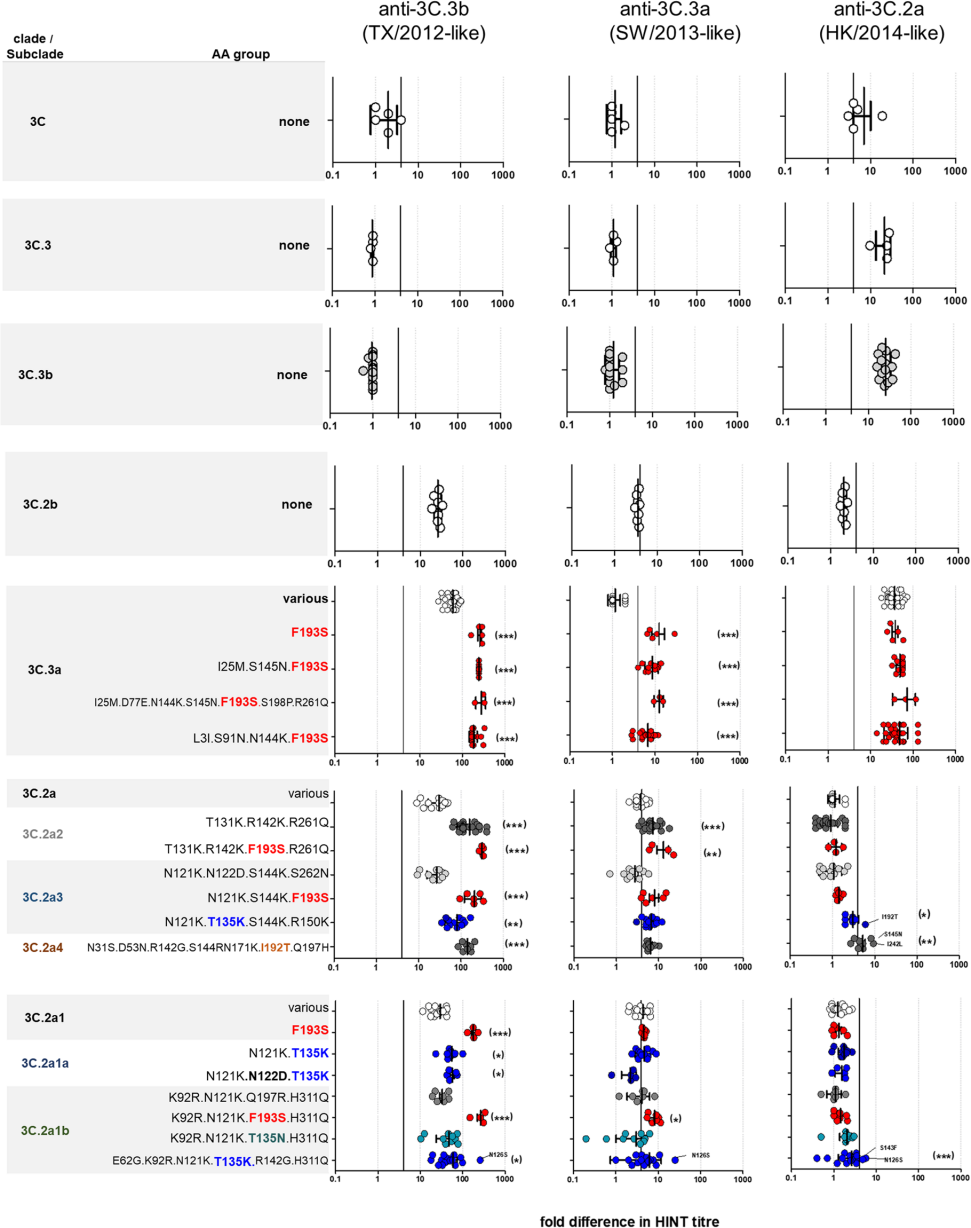
HINT neutralization profile of A(H3N2) viruses circulating during 2011–2018. HA clades (**A**) 3C, (**B**) 3C.3, (**C**) 3C.3b, (**D**) 3C.2b, (**E**) 3C.3a, (**F**) 3C.2a and (**G**) subclade 3C.2a1. HA1 AA changes compared to each HA clade consensus sequence are shown on the Y-axis. “Various” correspond to viruses that had a HA sequence identical to the clade-specific consensus sequence or viruses carrying AA substitutions that could not be assigned to any group. Genetic groups encoding substitutions at positions 135 and/or 193 are highlighted in bold blue and bold red, respectively. The vertical black line indicates a 4-fold reduction in HINT titre. Error bar indicates the population mean ± standard deviation of the mean. The *p* values were calculated using the nonparametric Kruskal-Wallis test with Dunns post-test. *p < 0.05, **p < 0.005, ***p < 0.0005 compared to group “various”.

HINT reference antisera were used to characterize 422 A(H3N2) viruses collected mainly in the U.S. during 2011–2018 (Figs 2 and S3), encompassing seven consecutive NH influenza seasons. These viruses were submitted for virological surveillance and underwent ≥1 passage(s) in cell cultures. Most viruses tested were cultured in SIAT1 cells, and those that were cultured in MDCK cells are denoted in the text. An effort was made to include viruses representing distinct genetic subclades and to incorporate viruses with HA AA substitutions previously shown or suspected to alter antigenic epitopes (e.g., substitutions at position 158 or 160). HA1 sequence analysis indicated that nearly all viruses tested in this study shared the AA substitutions Q33R, N145S and N278K (Fig. 1A), when compared to cell-propagated vaccine virus A/Victoria/361/2011 (clade 3C). Additionally, each phylogenetic clade possessed HA AA substitutions that were characteristic and that conferred specific antigenic properties, as described in detail below. Of note, all these signature changes were established by WHO CCs in previous years^25^.

#### Clade 3C viruses

Four MDCK-propagated clade 3C viruses collected during 2011–2012 were retrieved from the virus repository. These viruses contained NA mutants with AA substitutions at the residue 151 (Table S2), which contribute to NA-dependent viral attachment^26^. Therefore, testing was performed in the presence of the NA inhibitor oseltamivir to avoid interference of neutralisation of virus infectivity by anti-NA antibodies. While addition of oseltamivir produced no effect on the infected cell population (ICP) of the SIAT1-propagated reference viruses, it caused a drastic reduction in the number of cells infected by the MDCK-propagated viruses, including TX/2012 (Table S2). Overall, clade 3C viruses exhibited similar neutralization profile to A/Victoria/361/2011 and TX/2012 (Fig. 2A and Table S2).

#### Clade 3C.3 and 3C.3b viruses

Viruses belonging to clades 3C.3 and 3C.3b share T128A and R142G, both substitutions residing at antigenic site A (Fig. 1A). Substitution T128A has a similar effect to T128N present in A/Texas/50/2012 by causing a loss of an N-linked glycosylation (glc.) sequon at 126–128. Additionally, most viruses shared E62K (site E), N122D (glc.122 loss in site A), and L157S (site B); whereas a smaller set of viruses possessed T135K, which causes loss of glc. sequon at 133–135. A total of four viruses from clade 3C.3 and sixteen from clade 3C.3b were tested, and all showed the same neutralization pattern as the reference virus HI/34-3C.3b (Figs 2B,C and S3A), indicating they were antigenically similar to TX/2012. The viruses analysed were collected during 2014–2015 season; and ceased to circulate in the following seasons.

#### Clade 3C.3a viruses

**Figure 3:**
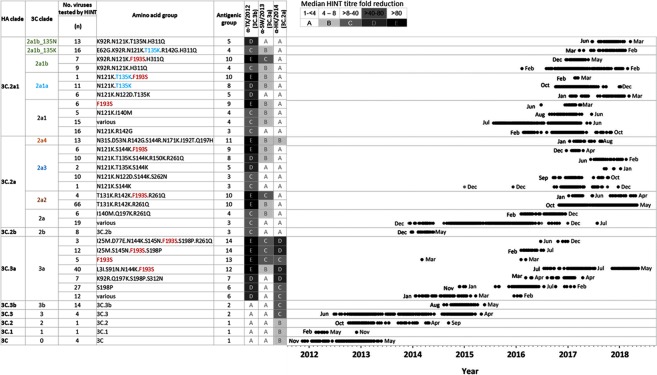
Antigenic Landscape using NGS and HINT data, 2011–2018. Neutralization profiles shown in Fig. 2 were separated in different antigenic groups according to HINT titre fold reduction as: (**A**) 1- to <4-fold, (**B**) 4- to 8-fold, (**C**) >8- to 40-fold, (**D**) >40- to 80-fold and (**E**) >80-fold. Based on this criteria, all viruses collected during 2011–2018 and tested using HINT fell into one of 14 antigenic groups (1–14). New nomenclature for clade 3C.2a subclades in shown next to the old nomenclature. To visualize the period of circulation of the different antigenic groups in U.S., the dates of virus collection were retrieved and plotted.

Viruses from clade 3C.3a share the same changes of T128A and R142G as seen in 3C.3 and 3C.3b but also share three additional HA markers: A138S (site A), F159S (site B) and N225D in the receptor binding site (RBS), with N225D also being present in viruses from clade 3C.2b and 3C.2a (Fig. 1A). A total of 106 clade 3C.3a viruses collected during 2014–2017 were tested (Figs 2E and 3). As expected, HINT analysis showed that all 3C.3a clade viruses were antigenically drifted from TX/2012 and HK/2014, showing a significant reduction in neutralization (>10-fold) (Fig. 2E). A group of viruses carried AA substitutions K92R, Q197K, S198P or S312N (Fig. 2E, group: various); however, these changes did not seem to affect neutralization by homologous or heterologous antisera compared to 3C.3a viruses having no such AA changes. Notably, nearly half of the viruses from clade 3C.3a characterized were antigenically distinguishable from SW/2013, exhibiting a > 4-fold reduction in neutralization by α-SW/2013-like antiserum (Fig. S3B). These viruses were collected during seasons 2015–2016 and 2016–2017, and all shared the AA substitution F193S (Fig. 2E). Moreover, the ability of α-TX/2012-like antiserum to neutralize these viruses was greatly diminished compared to group carrying F193 (designated as ‘various’). Residue 193 is part of the antigenic site B and is located on a highly exposed region within the 190-helix in RBS (Fig. S5A). Changes at this residue likely impact antibody recognition and promote escape. Frequently, viruses carrying F193S were found to have additional substitutions within other antigenic sites, such as N144K (site A), S145N (site A), S198P (site B) or R261Q (site E), pointing to a role for these substitutions in the observed phenotype. However, some viruses carrying F193S were antigenically distinguishable from SW/2013-like viruses but had no substitutions at either 144 or 145 (Fig. 2E, group: F193S). Taken together, these results indicate that F193S is likely to confer antibody escape from this clade-specific antiserum and antigenic distancing of 3C.3a viruses from SW/2013.

Interrogation of HA sequences revealed that 3C.3a viruses with F193S fell into three genetic subclades. The first subclade that shared the AA substitution I25M was detected in eight U.S. states from January to December, 2016. The second subclade shared the substitution L3I, was first detected in 2016, and has continued to circulate in other U.S. states and several countries in South and North Americas through 2018. All viruses from subclade I25M shared the substitutions S145N (site A) and S198P (site B), whereas viruses from the subclade L3I shared the substitutions S91N (site E) and N144K (site A), which causes loss of glc. sequon (144–146). These two subclades had very similar HINT profiles, suggesting that N144K did not further impact ferret serum recognition. Finally, the third group of viruses detected from October to December 2016, carried the substitutions I25M.D77E.N144K.S145N.F193S.S198P.R261Q and exhibited 10-fold reduction in neutralization by SW/2013-like antiserum (Fig. 2E).

#### Clade 3C.2b viruses

Clade 3C.2b comprised a small group of viruses that circulated in the U.S. and several other countries for a short period of time during the 2013–2014 season (Fig. 3). Viruses belonging to this group acquired the substitution D53N (site C) and shared the following markers with clades 3C.2a and 3C.2a1: L3I, N144S (site A), K160T (site B), N225D (RBS) and Q311H (Fig. 1A). In contrast to clade 3C.2a and subclade 3C.2a1, clade 3C.2b viruses did not carry the substitution F159Y (site B). Residue 159 is located on a highly exposed region of antigenic site B (Fig. S5B), and viruses with the substitutions F159Y or F159S have antigenically drifted away from TX/2012^12^.

Clade 3C.2b viruses were retrieved from the virus repository and were previously propagated in standard MDCK cells acquiring substitutions in their HA and NA sequences not found in the matching clinical specimens (Table S3). Of eight MDCK isolates tested, two contained mixtures of variants carrying HA AA substitutions that caused a loss of a glc. sequon at 158–160, while five isolates showed either the presence of NA-attachment mutants with substitutions at 151, or had T148K that abolishes a glc. sequon at 146–148 in the NA^26,27^ (Table S3). As the matching clinical specimens were available, we used them to re-isolate viruses in SIAT1 cells. NGS analysis confirmed the lack of substitutions in HA and NA genes of the newly isolated viruses, and upon testing using HINT, all eight SIAT1 isolates shared a similar neutralization profile (Fig. 2D, Table S3). This data provides additional evidence demonstrating the rapid selection of mutants in standard MDCK cells, and underlines the importance of utilizing SIAT1 cell line for isolation of contemporary influenza H3N2 viruses.

The HINT profiles of clade 3C.2b viruses showed that they were very antigenically distinct from TX/2012 (19-34-fold down) and similar to HK/2014 (2-fold down), despite lacking the F159Y^12^. Clade 3C.2b viruses also showed a 3-4-fold reduced neutralization by the α-SW/2013-like serum (Fig. 2D, Table S3). Our results demonstrate that the gain of a glycan at 158 was the key molecular determinant of the antigenic distancing of clade 3C.2b (as well as clade 3C.2a and subclade 3C.2a1) viruses from TX/2012.

#### Clade 3C.2a viruses

Viruses from clade 3C.2a were first detected in December 2013 and have become the dominant A(H3N2) virus group in circulation in U.S. over several seasons, including the high severity 2017/18 NH season^2^. Both, clade 3C.2a and subclade 3C.2a1 share the substitution F159Y, not found in clade 3C.2b viruses (Fig. 1A). Interrogation of the HA sequences showed the existence of various AA groups (Fig. 2F) co-circulating during 2014–2018 (Figs 3 and S3C). HINT analysis indicated that all 3C.2a viruses were antigenically different from TX/2012, showing poor neutralization by the α-TX/2012-like serum (Fig. 2F), which is consistent with other studies^12,22–25^. Approximately one quarter of viruses characterized exhibited ≥30-fold reduction in neutralization by the α-TX/2012-like serum, while the remaining viruses exhibited ~100-fold drop in titre (Fig. 2F). Viruses exhibiting 30-fold reduction in titre that circulated December 2013 to December 2016, belonged to the groups annotated as “various” or “2a3” (Fig. 2F).

Clade 3C.2a was recently divided by WHO CCs into new subclades^3^, and for convenience we present the HINT data according to this classification below:

- **2a2**. This group of viruses carried the AA substitutions T131K.R142K.R261Q and showed diminished neutralization by α-SW/2013-like serum and little or no neutralization by α-TX/2012-like serum (Fig. 2F). Interestingly, the presence of the substitutions T131K (site A), R142K (site A), and R261Q (site E) did not seem to affect neutralization by α-HK/2014-like serum. 3D structural analysis using PyMOL, showed that residues 131 and 142 are located on a highly exposed region on the rim of RBS, whereas AA 261 is distal and located on the lateral side of the HA trimer (Fig. S5C). Thus, we speculate that T131K and R142K, rather than R261Q, have a stronger impact on recognition by α-SW/2013-like serum. In addition, a subset of viruses acquired F193S and exhibited further diminished neutralization by α-SW/2013-like and α-TX/2012-like sera, although this difference was not significant when compared to AA group T131K.R142K.R261Q, likely due to the already limited neutralization exhibited by this group (Fig. 2F). This data indicates that substitutions T131K.R142K.R261Q and F193S contribute to the antigenic distancing of 3C.2a viruses from TX/2012 and SW/2012, but not from HK/2014.
- **2a3**. These viruses could be separated in three AA groups: (1) N121K.N122D.S144K.S262N that exhibited ~30-fold reduction in neutralization by α-TX/2012-like serum, and ≤4-fold reduction in neutralization by α-SW/2013-like serum; (2) N121K.S144K.F193S that exhibited ≥100-fold reduction in neutralization by α-TX/2012-like serum and ≥4-fold reduction in neutralization by α-SW/2013-like serum; and 3) N121K.T135K.S144K.R150K that displayed ~60-fold and ≥4-fold reduction in neutralization by α-TX/2012-like and α-SW/2013-like sera, respectively. Although, all these viruses were neutralized by α-HK/2014-like serum (<4-fold titre drop), group 3 was significantly less inhibited than group 1 (p = 0.0001) and group 2 (p = 0.0006). Only one virus from group 3 that carried the additional substitution I192T displayed a 6-fold reduction in neutralization by α-HK/2014-like serum.
  Viruses from group N121K.N122D.S144K.S262N carried N122D, which causes a loss of glyc. sequon at 122–124 in site A, and were antigenically indistinguishable from those with N122, suggesting that this site does not significantly contribute to recognition by ferret antibodies elicited by infection with recent H3N2 viruses.
  In addition, group N121K.T135K.S144K.R150K, carried the substitution T135K that causes the loss of glc.133–135 sequon. 3D structural visualization indicated that residue 135 is in close proximity to residue 144 within the RBS, whereas AA 150 is located proximal to 121 and 261, on the lateral side of the HA trimer (Fig. S5C). Of note, the four AA substitutions in this group involve the gain of a Lys residue, which likely contribute to increasing the positive charge of the HA surface.
- **2a4**. Viruses from this subclade shared N31S.D53N.R142G.S144R.N171K.I192T.Q197H and exhibited reduced neutralization by α-TX/2012-like and α-SW/2013-like sera. Notably, these viruses displayed 3- to 10-fold reduction in neutralization by α-HK/2014-like serum, providing some evidence of antigenic distancing from HK/2014 (Fig. 2F). The Ile → Thr substitution affecting residue 192, which is located on the 190-helix of the RBS and in antigenic site B (Fig. S5C), combined with R142G and/or S144R are likely responsible for immune escape, although we cannot rule out the contribution of the substitutions N31S, D53N and N171K.

Overall, the majority of clade 3C.2a viruses were antigenically similar to HK/2014 with the exception of a small group of viruses from subclade 2a4 that exhibited a limited degree of antigenic distancing. Viruses from this subclade have not been detected since December 2017^3^.

#### Subclade 3C.2a1

Viruses from subclade 3C.2a1 branched out of clade 3C.2a in 2015 and have been in circulation since then. These viruses shared the markers L3I.N144S.F159Y.K160T.N225D.Q311H with clade 3C.2a, while carrying the characteristic HA1 AA substitution N171K (site D).

Sequence analysis of HA showed the existence of at least eight AA groups (Fig. 2G) co-circulating during 2015–2018 (Fig. 3). A large set of 3C.2a1 viruses carried the substitutions N121K (site D) and R142G (site A). Overall, the majority of subclade 3C.2a1 viruses were antigenically similar to HK/2014, as previously observed by WHO CC analyses^4^.

Subclade 3C.2a1 has branched into new subclades^3^, and for convenience we present the data according to this classification below:

- **2a1**. These viruses could be separated in three groups: (1) viruses that carried the HA consensus sequence for subclade 3C.2a1; (2) viruses with AA substitutions that were not characteristic of other subclades (i.e. N121K and R142 G), but did not affect the antigenic properties of the viruses; or 3) viruses that carried the AA substitution F193S (Fig. 2G). HINT analysis indicated that 2a1 viruses were antigenically distinguishable from TX/2012 and SW/2013. They exhibited ~30-fold reduction in neutralization by α-TX/2012-like serum and ~4-fold reduction in neutralization by α-SW/2013-like serum (Figs 2G and S3D). Interestingly, viruses that carried the F193S exhibited >100-fold reduction in neutralization by α-TX/2012-like serum, a phenotype similar to that of clade 3C.2a and clade 3C.3a viruses carrying the same substitution.
- **2a1a**: Viruses from this subclade shared N121K.T135K or N121K.N122D.T135K (Fig. 2G). T135K causes a loss of glc. sequon at position 133–135 (site A), while N122D is predicted to cause loss of glycan at the 122–124 position in HA. Both groups exhibited a ~60-fold reduction in neutralization by α-TX/2012-like serum. Interestingly, viruses carrying K135 were neutralized to a similar degree as those carrying T135 by α-SW/2013-like serum (~4-fold drop), whereas the set of viruses carrying T135K in addition to N122D exhibited slightly improved neutralization (p = 0.0036) compared to those carrying T135K only by this clade-specific antiserum. It is likely that loss of two glc. sequons at antigenic site A may expose an epitope originally shielded by these glycans, providing better access for antibodies directed against 3C.2a-like viruses.
- **2a1b**. These viruses share the substitutions K92R.N121K.H311Q (Fig. 2G), and are estimated at a global frequency of ~30% (as of August 2018), with particularly higher frequency in Asia^3^. Subclade 2a1b could be separated in four groups: (1) K92R.N121K.Q197R.H311Q that exhibited ~30-reduction in neutralization by α-TX/2012 serum, (2) K92R.N121K.T135N.H311Q and (3) E62 G.K92R.N121K.T135K.R142 G.H311Q, both exhibited a ~60-reduction in neutralization by α-TX/2012 serum; and (4) K92R.N121K.F193S.H311Q that displayed a >100-fold reduction in neutralization by α-TX/2012-like serum. All these viruses displayed ~4-fold reduction in neutralization by α-SW/2013-like antiserum, expect for those carrying F193S that exhibited a >4-fold reduction.

Substitution T135N causes loss of glc.133–135 sequon and creates a new predicted glc. sequon at residues 135–137. Although, these substitutions seemed to slightly reduce neutralization of 2a1b viruses by TX/2012-like and HK/2014-like antisera (Fig. 2G, subclade 2a1b.T135N), the difference in neutralization was not statistically significant (p > 0.05) when compared to the viruses carrying T135 (Fig. 2G, 2a1-group various).

Notably, the group of viruses that share E62G.K92R.N121K.T135K.R142G.H311Q (Fig. 2G, subclade 2a1b.T135K) exhibited a ~3-fold reduction in neutralization by α-HK/2014-like antiserum, with a couple of viruses carrying the additional substitutions S143F (site A) or N126S (glc.126 loss at site A) exhibiting a > 4-fold reduction in neutralization by α-HK/2014-like serum. 3D structural analysis using PyMOL showed that residue 135 is in very close proximity to residues 142 and 143 on the rim of the RBS, residues 62 and 92 are adjacent to one another and located on the opposite lateral side of the HA trimer to residues 121, 126 and 311 (Fig. S4D).

Taken together, these data indicate that the vast majority of 3C.2a1 viruses tested in this study were antigenically indistinguishable from HK/2014, however a small number of viruses from subclade 2a1b.T135K, seen in season 2017–2018, were antigenically distinct.

### Antigenic Cartography

**Figure 4:**
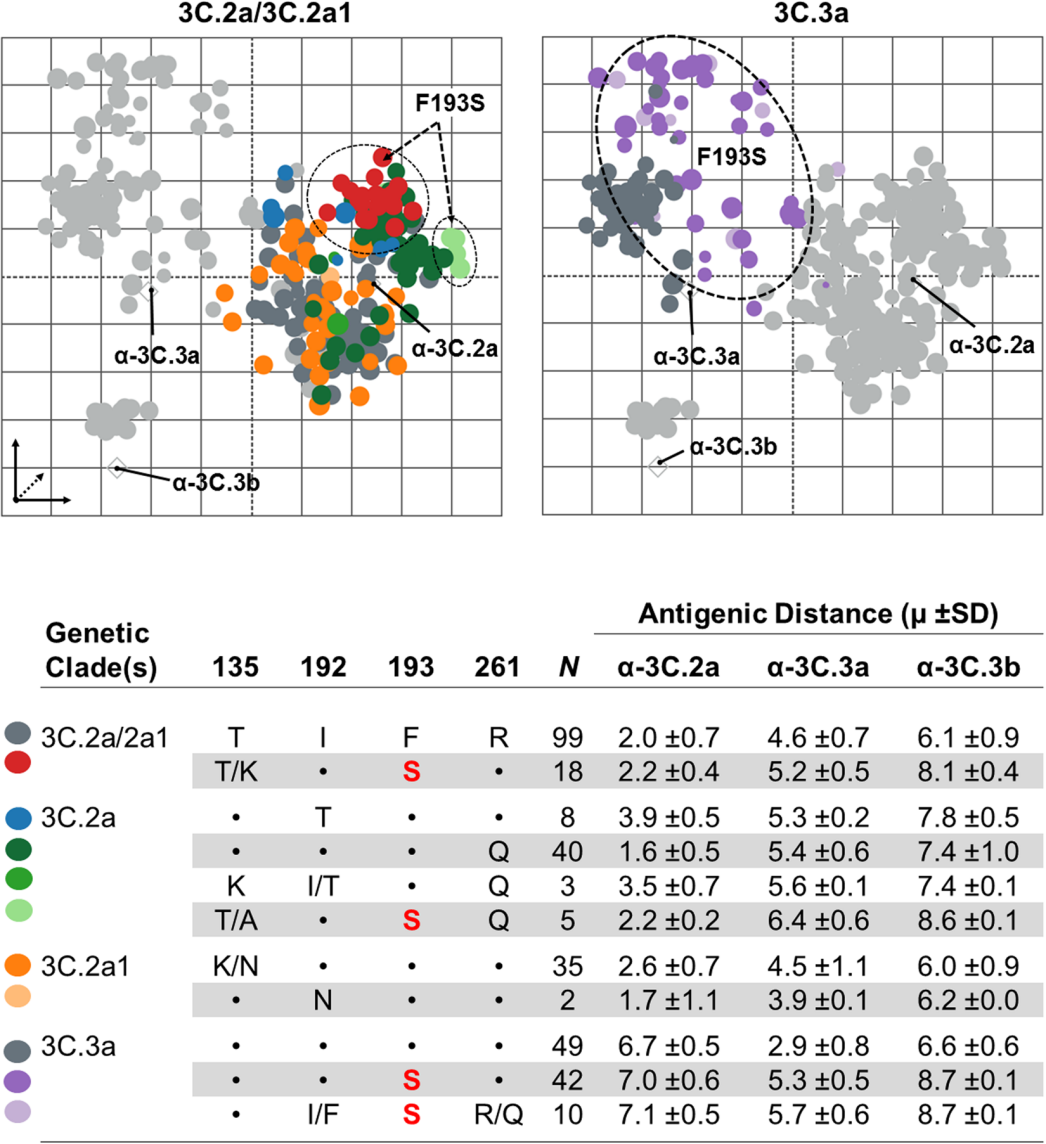
Modelling antigenic trends in the A(H3N2) virus HINT landscape. Genetic identities at positions 135, 192, 193, and 261 in the HA protein are overlaid on antigenic maps for (**A**) 3C.2a/2a1 and (**B**) 3C.3a strains with mean (±SD) antigenic distance relative to (**C**) α-3C.2a (AK/140), α-3C.3a (MA/14), and α-3C.3b (HI/34) antisera. Dashed lines in Panels A and B, highlighted 3C.2a/2a1 and 3C.3a protein sequences which encode the F193S substitution (bold red in Panel C). Dots in Panel C, no sequence difference from the 3C.2a consensus. Light gray icons, strains which encode (**A**) non-3C.2a/2a1 and (**B**) non-3C.3a protein sequences. 3C.3 (*N* = 4), 3C.2b (*N* = 8), and 3C.3b (*N* = 14) strain comparisons are not shown. Antigenic map parameters are as detailed in Fig. S4.

We applied the ACMACS antigenic cartography algorithm (adapted from^5^) to provide a computational framework and analyse linked specimen metadata (*e*.*g*., genetic clade, HA protein sequence, etc.) to modelled antigenic trends. Spatial relationships for multiple virus-antiserum reactivities in 2014–2018 surveillance specimens were effectively modelled in multi-dimensional landscapes to assess antigenic relatedness or drift between applied viruses and reference vaccine antisera. From this perspective, HINT antigenic trends were optimized in 5D cartographic space with applied A(H3N2) strains clustering into clade-specific groups: (1) 3C.2a/3C.2a1/3C.2b, (2) 3C.3a, and (3) 3C.3/3C.3b based on reactivity to α-HK/2014-like, α-SW/2013-like, and α-TX/2012-like sera, respectively (Figs 4 and S4). Mean Euclidean distances in antigenic units (AU) [*n* AU = 2^*n*^-fold titre drop] within these groups were: 2.2 ± 2.2 AU for HK/2014-like, 4.1 ± 1.4 AU for SW/2013-like, and 1.3 ± 0.2 AU for TX/2012-like; with aggregate reactivities to antisera outside these groups ranging from 3.2 AU to 7.7 AU (Fig. 4C)

Overlay of HA protein sequence data onto the HINT multi-dimensional antigenic landscape provided evidence of antigenic distancing within the α-HK/2014-like and α-SW/2013-like groups by HINT (Fig. 4). Linkage of AA identities at four previously defined positions: 135, 192, 193, and 261 in the HA protein identified >10 unique protein sequences compared to the HK/2014-like consensus sequence (T135.I192.F193.R261) (Fig. 4C). In particular, the F193S substitution alone or combined with I192F. R261Q, in clade 3C.3a specimens resulted in a ≥2.4 AU and 2.1 AU increased antigenic distancing from α-SW/2013-like and α-TX/2012-like antisera, respectively. Furthermore, the F193S substitution in 3C.2a and 3C.2a1 isolates also conferred increased distance to α-TX/2012-like serum despite negligible reactivity differences to either α-HK/2014-like or α -SW/2013-like sera.

Collectively, application of the ACMACS algorithm to HINT data and linked metadata provides evidence of detectable antigenic distancing within 2014–2018 3C.2a, 3C.2a1, and 3C.3a viruses to concurrent, representative A(H3N2) vaccine strains (cell-propagated vaccine virus counterparts).

### Antigenic landscape of A(H3N2) viruses circulating in U.S., 2011–2018

To represent the antigenic profiles of viruses collected during 2011–2018 in a heatmap format, HINT titre folds were converted to Log_2_ creating up to five categories per antiserum: (A) <2, (B) 2 to <3, (C) 3 to <5, (D) 5 to <7, and (E) ≥7 (Fig. 3). Combining heatmaps for all three reference antisera yielded 14 antigenic groups (Fig. 3). Some HA clades (e.g. 3C.3 and 3C.3b) were represented by a single antigenic group, while others had several antigenic groups (e.g. clade 3C.2a). Notably, the vast majority of influenza A(H3N2) viruses tested in this study were effectively neutralized (<4-fold titre drop) by at least one of the three reference antiserum, with the exception of clade 3C.3a viruses carrying F193S.

To visualise the period of circulation of the different antigenic groups in the U.S., the dates of virus collection were incorporated (Fig. 3). This included all viruses affiliated with an antigenic group based on their HA clade and genetic markers, which encompassed viruses tested using HINT. This illustrates viruses from different HA clades co-circulated within the same season (Fig. 3). For example, in January 2017, there were three clades (3C.3a, 3C.2a, and 3C.2a1) represented by at least 13 antigenic groups in circulation among people.

Substitution F193S, which caused antigenic distancing was detected in some viruses from clade 3C.3a circulating in 2014, and then it was seen again among viruses from clades 3C.3a, 3C.2a and subclade 3C.2a1 during 2016–2018. Within clade 3C.2a, subclade 3C.2a2 (group T131K.R142K.R261Q) was detected in 2016 and it has been the predominant group in 2017–2018 season. On the other hand, subclade 3C.2a4 (N31S.D53N.R142G.S144R.N171K.I192T.Q197H) exhibited reduced neutralization by α-HK/2014 serum was detected since January 2017; however, after August 2017 it appears to be on decline^3^.

Within subclade 3C.2a1, 2a1b viruses have increased rapidly in detection in fall 2017 and are still detected in circulation^3^. Notably, some viruses carrying T135K (Fig. 2G, group E62G.K92R.N121K.T135K.R142G.H311Q) showed some antigenic distance from HK/2014.

These data indicate that, although there were some antigenic groups of concern, most of the viruses tested showed less than 4-fold reduction in neutralization by at least one antiserum representing the cell-propagated vaccine viruses TX/2012, SW/2013, and HK/2014.

### Effect of N-linked glycosylation at residue 158

**Figure 5:**
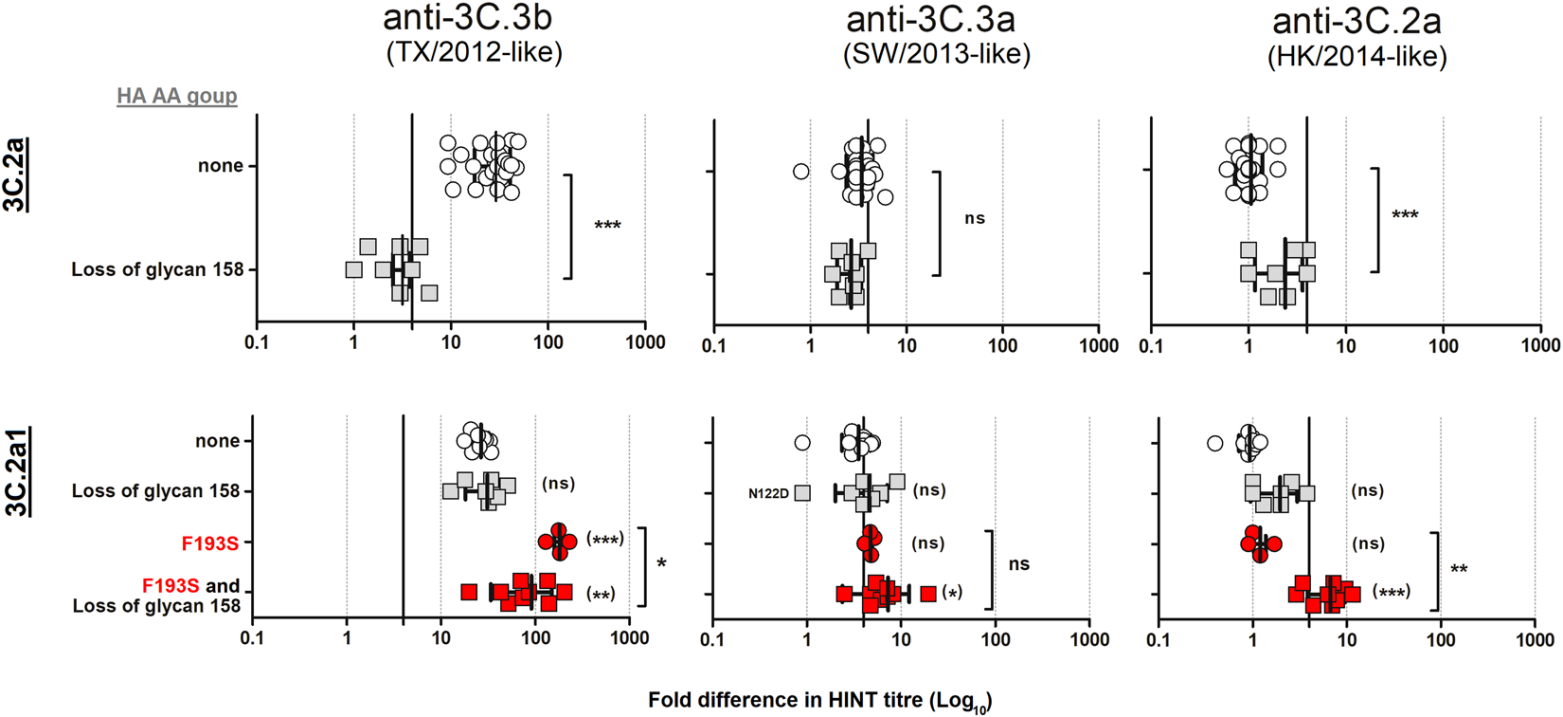
Effect of glycosylation sequon 158–160 loss on HINT neutralization patterns for 3C.2a and 3C.2a1 viruses. HA (**A**) clade 3C.2a and (**B**) subclade 3C.2a1. HA1 AA changes compared to each HA clade consensus sequence are shown on the Y-axis. Genetic groups which encode the F193S substitution are indicated in bold red. The vertical black lines indicate a 4-fold reduction in HINT titer. The bar indicates the mean ± standard deviation of the mean. The *p* values were calculated using a *Paired t test*. Ns = no significant; *p < 0.05, **p < 0.005, ***p < 0.0005.

It was previously recognized that some viruses upon isolation in cell culture acquired changes at either residue 158 or 160, not found in the matching clinical specimens, predicted to cause the loss of a N-linked glycan. It was reported by WHO CCs and others^10,28^. Therefore, all clade 3C.2a and 3C.2a1 isolates tested in this study (n = 284) were analysed for sequence polymorphism at nucleotides encoding the glc. sequon N_158_-X-T_160_. The majority of clade 3C.2a (140/162) and subclade 3C.2a1 (106/122) isolates retained this glc. sequon, and the HINT results for those isolates that exhibited loss of glc.158 were excluded from the data (Figs 2 and 3). A group of clade 3C.2a isolates were found to contain mixtures with over 50% of the HA variant lacking glc.158. These viruses displayed a significantly improved neutralization by α-TX/2012-like serum compared to viruses that had glc.158, whereas they exhibited slightly reduced neutralization by α-HK/2014-like serum (Fig. 5A). This result suggests that the presence of glc.158 alone was sufficient to cause escape from antibodies in α-TX/2012 sera, further strengthening the notion that glycan 158 was likely responsible for the observed antigenic drift of clade 3C.2b viruses from TX/2012.

Subclade 3C.2a1 viruses that lost glc.158 showed a very similar neutralization profile to those with glc.158 by α-TX/2012-like and α-SW/2013-like sera, and slightly reduced neutralization by α-HK/2014-like antiserum (Fig. 5B). However, when glc.158 loss occurred in viruses carrying the substitution F193S, the absence of glycan at 158 significantly improved neutralization by α-TX/2012-like antiserum, yet it impaired neutralization by α-SW/2013-like and α-HK/2014-like sera.

Taken together, this data provides more evidence regarding the selection of variants with culture adaptation, and emphasises the importance of close monitoring of HA sequence during influenza H3N2 viruses culturing prior to antigenic analysis.

### Testing viruses in human respiratory specimens

**Figure 6:**
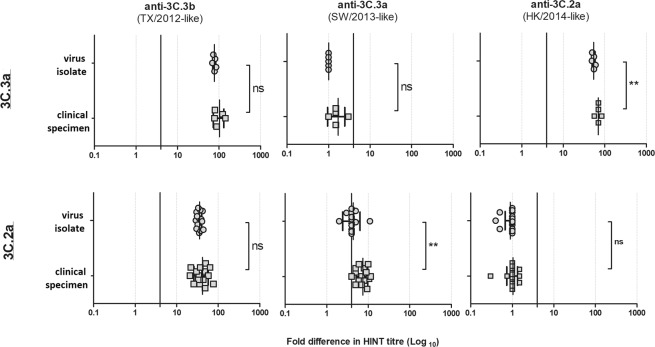
HINT neutralization profile of clinical specimens and matching virus isolates collected in the 2014–2015 season. HA clades (**A**) 3C.3a and (**B**) 3C.2a. The vertical black lines indicate a 4-fold reduction in HINT titre. The bar indicates the mean ± standard deviation of the mean. The *p* values were calculated using a *Paired t test*. ns = no significant; *p < 0.05, **p < 0.005, ***p < 0.0005.

Finally, we sought to explore the applicability of HINT to directly test viruses in human specimens. A total of 20 residual human respiratory specimens positive for A(H3N2) contained sufficient quantity of infectious virus (≥6 × 10^3^ IU/ml) to be tested with the three antisera. The neutralization profiles of clade 3C.3a (n = 5) (Fig. 6A) and clade 3C.2a specimens (n = 15) tested, (Fig. 6B) had a very similar neutralization profile to that of their respective clade-specific reference virus (see Fig. 2E,F, group various). These results provide proof that HINT can be used to directly characterize virus in clinical specimens, however this may not always be feasible due to the low quantity of infectious virus particles in aliquots submitted for virological surveillance. Thus, as an alternative we evaluated whether propagation of clinical specimens in MDCK-SIAT1 cells for a short period (24 hrs) would sufficiently increase virus titres while maintaining the original antigenic properties of the viruses. Overall, the HINT neutralization patterns of the isolates were comparable to those of their matching clinical specimens (Fig. 6).

Interestingly, when we compared matching clinical specimens and isolates carrying identical HA and NA protein sequences, we observed that MDCK-SIAT1-isolates were somewhat more efficiently neutralized (higher HINT titres) by the reference antisera than clinical specimens (p < 0.05; Fig. 6A, 3C.3a viruses neutralized by 3C.2a-antiserum and Fig. 6B, 3C.2a viruses neutralized by 3C.3a-antiserum). We believe this variance may reflect some differences at host-specific post-translational modification of the virus surface proteins (i.e. complexity of glycosylation patterns), however, further studies would be needed to fully understand the molecular mechanism.

## Discussion

The diminished ability of recent influenza A(H3N2) viruses to agglutinate RBCs precludes them from being antigenically characterized using HI assay. Therefore, in recent years alternative assays have been developed and implemented. The purpose of this study was to develop an optimized neutralization assay (HINT) to assess antigenic relatedness of influenza A(H3N2) viruses. HINT and the reference viruses and antisera were designed with the intent to reduce antigenic mischaracterization deriving from virus adaptation to cell culture.

The use of traditional neutralization assays can be challenging due the variance in replication kinetics of different influenza viruses. The optimal ratio between the number of cells, dilution of virus, concentration of trypsin, and incubation time, must be empirically determined^18,29^. In our study, trypsin was omitted and inoculum was set 150–6,000 infectious units per well, which resulted in consistent infection and reproducible results. By omitting trypsin, we also eliminated the need for a semi-solid overlay (e.g. avicel, agar, or carboxymethyl cellulose) to restrict virus spread to neighbouring cells. Under these conditions, the infected cell population (ICP) is limited to those cells infected by the residual non-neutralized virus; hence, it is independent of the kinetics of virus replication.

Different technologies have been described to detect and count ICP^18^. Here, Hoechst 33342 dye and NP-immunostaining were used to identify infected cells, followed by a microplate reader with a high-content imaging capability and the Gen5ImagePlus software to aid microscopic image analysis. HINT titres were determined by curve-fitting analysis by estimating the reciprocal dilution of antiserum at which ICP was reduced by 50% (IC_50_). Other neutralization assays have been optimized to a cut-off of 80%^18^ or ≥90%^29^. IC_50_ may be more suitable in instances when antiserum displays low homologous titres, and when a 90% reduction in ICP may not be achievable even at the lowest feasible antiserum dilution.

In this study, reference antisera were generated by infecting ferrets with human respiratory specimens. The quality of the antisera determines the ability of the neutralization assay to reliably discriminate between these antigenic groups and, ultimately, to detect further antigenic distancing. We rationalized that using clinical specimens instead of cell- or egg-cultured viruses would prevent eliciting antibodies against HA epitopes that might acquire adaptive substitutions during virus propagation in culture. The chosen respiratory specimens contained viruses representing the three major antigenic groups that co-circulated during 2014–2015, namely, TX/2012-like, SW/2013-like and HK/2014-like viruses. Interestingly, ferrets inoculated with the SW/2013-like virus, MA/14-3C.3a, developed low homologous HINT titres, and the antisera from all four animals showed low ability to discriminate between homologous and heterologous viruses. This result is consistent with a previous report, where SW/2013 was used to raise post-infection antiserum^12^. Chambers *et al*. showed that F159S (characteristic of clade 3C.3a) resulted in an asymmetrical antigenic change, in which serum antibodies directed against the vaccine virus SW/2013 neutralized TX/2012 (which carried F159) as homologous, but antibodies raised against TX/2012 reacted poorly towards SW/2013 and other viruses carrying F159S^12^. This pattern of reactivity was previously identified during the WHO Vaccine Candidate Selection Meeting in September 2015^30^. Consistently, our results showed that α-TX/2012 serum poorly neutralized MA/14, but α-MA/14 serum neutralized TX/2012 as the homologous virus.

The reference virus HI/34-3C.3b was antigenically similar to TX/2012, and as α-TX/2012 serum, the antiserum raised against HI/34 poorly neutralized clades 3C.2a and 3C.3a reference viruses^12^. The antiserum raised against AK/140-3C.2a, a HK/2014-like virus, poorly reacted with other reference viruses.

The HINT reference panel was used to analyse the antigenic relatedness and immune escape of A(H3N2) viruses co-circulating during seven consecutive seasons, 2011–2018. Although the study was focused on characterizing virus isolates, a set of clinical specimens was successfully examined as well, paving the way to further improvement of antigenic analysis. Viruses characterized in this study belonged to seven HA phylogenetic clades. When a >4-fold drop in titre was detected, that virus was considered antigenically different from a homologous reference virus (or a vaccine strain). It would be prudent to monitor spread of such viruses, even if 4-fold titre drop may not manifest an antigenic drift. Noteworthy, the previously shown antigenic drift viruses tested here using anti-TX/2012 serum displayed >8 fold reduction in neutralization.

Using the reference antisera representing the antigenic groups circulating during 2014–2015, TX/2012-, SW/2013- or HK/2014-like viruses and introducing arbitrary criteria to rank results of HINT (from <4 to >80-fold drop), we were able to sort all tested viruses into 14 distinct antigenic groups. Some clades, such as 3C.3, 3C.3b, and 3C.2b contained a single antigenic group, whereas other clades (e.g. 3C.2a) exhibited notable antigenic diversity. Clades 3C.3a and 3C.2a, which drift from TX/2012, shared the HA AA substitutions F159S/Y and N225D^12^. Li *et al*. demonstrated that viruses carrying N225D.F159S (present in 3C.3a) or N225D.F159Y (present in 3C.2a) were among the most frequent TX/2012 escape mutants selected by human antisera^31^. It is possible that N225D improved receptor binding and compensated for F159Y/S, occurring at antigenic site B. Acquisition of F159S or F159Y were shown to play a critical role in antigenic drift from TX/2012^12^. Hence it was surprising that clade 3C.2b viruses, which carried F159, exhibited a HINT profile very similar to viruses from clade 3C.2a (with F159Y). Notably, clade 3C.2b and 3C.2a viruses shared a glycosylation sequon N_158_-X-T_160_ located on the tip of HA (antigenic site B). Our data indicated that glycan gain at 158 (illustrated by similar patterns in viruses with substitutions at either 158 or 160) was the most likely cause of antigenic distancing from TX/2012. With the concurrent extinction of clade 3C.2b viruses and rise of the now dominant 3C.2a clade, it is tempting to speculate that F159Y provided a fitness advantage, improving receptor binding and compensating for the presence of the bulky glycan 158 near the RBS.

Previous reports have shown that cultured viruses can lose the glycosylation sequon N_158_-X-T_160_, a phenomenon that we observed through the course of this study. Glycan loss was more common among MDCK-propagated viruses; however, some viruses showed evidence of loss after culturing in MDCK-SIAT1 cells, the preferred cell line for growth of contemporary A(H3N2) viruses. In clade 3C.2a viruses, glc.158 loss resulted in significantly improved neutralization by α-TX/2012-like serum, and slightly reduced neutralization by α-HK/2014-like serum. Consistent with our results, Zost *et al*. demonstrated that antisera raised against egg-adapted HK/2014-like vaccine virus carrying the T160K substitution failed to neutralize clade 3C.2a viruses glycosylated at residue 158^10^. In subclade 3C.2a1, glc.158 loss also caused improved neutralization by α-TX/2012-like serum and reduced neutralization by α-HK/2014-like antiserum, but only when viruses carried F193S.

The presence of frequent substitutions at HA residues 121, 122, 135, 144, 145, 150, and 193, among others, was observed in different clades. Notably, the parallel substitution F193S was found in clades 3C.3a, 3C.2a, and subclade 3C.2a1 where it affected neutralization by α-TX/2012-like and α-SW/2013-like sera. Antigenic cartography confirmed that viruses encoding F193S were antigenically separated from TX/2012-like and SW/2013-like viruses. 3C.3a viruses encoding F193S exhibited distinct clustering patterns separated from the main group; in addition, 3C.2a and 3C.2a1 viruses carrying F193S exhibited increased distance from α-TX/2012-like and α-SW/2013-like sera compared to counterparts encoding the HK/2014-like F193 consensus allele. Substitution F193S has been detected in 4.31% of all HA sequences submitted to Flusurver^32^ being first detected in February 2014 in the A(H3N2) virus A/Seoul/APD82/2014, and most recently detected in January 2018 in the A(H3N2)v virus A/swine/Iowa/A02139353/2018. Residue 193 is positioned adjacent to residue 159 on the membrane distal periphery of the RBS in a highly exposed region under positive immune selection^33^. Consistent with our data, Koel *et al*. showed that substitutions affecting residue 193 can cause cluster transition, due in part to the key position of this residue^34^. Moreover, a recent study demonstrated that TX/2012-like viruses carrying F193S were among the escape mutants selected by post-infection human antisera, highlighting the role of this substitution in antigenic drift^31^.

Clades 3C.3 and 3C.3b viruses were antigenically indistinguishable from TX/2012, exhibiting no antigenic diversity according to the HINT results. These viruses were replaced by clade 3C.3a that started circulating in January 2014 and was antigenically distinguishable from TX/2012. Acquisition of F193S by clade 3C.3a viruses led to antigenic distancing from SW/2013 and further distancing from TX/2012. The majority of 3C.3a viruses circulating since 2017 belonged to the antigenically distinct clade L31I.S91N.S144K.F193S.

Clade 3C.2a appears to exhibit higher genetic diversity than clade 3C.3a. Antigenically, clade 3C.2a viruses were distinguishable from TX/2012- and SW/2013. At the time when SW/2013 was selected by WHO CCs as the vaccine virus, ferret antisera raised against this virus inhibited well a majority of circulating viruses including those from clade 3C.2a. Some evidence of antigenic distancing from the HK/2014 was observed in viruses from subclade 3C.2a3 that acquired T135K, and subclade 3C.2a4 (carrying I192T). The recent increase in global frequencies of clade 3C.2a3 is attributed to the gain of T135K^3^. Subclade 3C.2a4 was elevated frequency in Oceania in July 2017 followed by a rapid decline in circulation^3^. It is worth mentioning that predominant subclade (3C.2a2) circulating in the US during season 2017–18 did not display signs of antigenic distancing from HK/2014.

As their predecessors, subclade 3C.2a1 viruses were antigenically distinguishable from TX/2012 and SW/2013, and were efficiently neutralized by α-HK/2014-like serum. Only a small set of viruses from subclade 3C.2a1b showed slightly reduced neutralization by α-HK/2014-like serum. This subclade has exhibited a rapid increase in global frequency at the end of the 2017 Southern Hemisphere season, and during the 2017–18 Northern Hemisphere persisted at lower frequency^3^.

In seasons 2016–2017 and 2017–2018, HA substitutions affecting residue 135 were frequently detected. T135K was often accompanied by substitutions at residues 121, 122, 142, 144 or 150. All these residues, except for 121, are in antigenic site A. Previous reports have suggested that substitutions associated with major antigenic transitions are located exclusively in HA antigenic sites A and B^34^. T135K is predicted to cause loss of the glycosylation sequon 133–135, while T135N is predicted to cause shift of glycan 133 to position 135.

Notably, many viruses tested in this study were antigenically indistinguishable from the reference viruses, despite possessing AA substitutions previously detected in mutants that escaped recognition by either monoclonal antibodies or human sera^5,31,35,36^. It is likely that our panel of three reference ferret antisera recognize a portion of the many epitopes that contribute to antigenic drift of seasonal viruses in a human host. Alternatively, it is possible that this discrepancy reflects the difference on epitope immunodominance between ferrets and humans. Another limitation of the study is that egg-cultured vaccine viruses (and the respective antisera) were not analysed, since our focus was to mitigate artifacts of virus culturing on antigenic analysis. The loss of glycan 158 and/or substitution L194P are characteristic changes seen in egg-propagated vaccine viruses that alter HA antigenic properties^10^. The effect of these changes on antibody recognition was beyond the scope of this study, however it will be addressed in future studies.

In summary, by utilizing HINT and our reference material we provided further understanding of antibody escape by A(H3N2) viruses co-circulating from 2011–2018. HINT and NGS analysis indicate that the majority of viruses circulating during 2016–2018 were efficiently neutralized by antisera raised against SW/2013- and HK/2014-like viruses. This data is consistent with the WHO influenza virus recommendations, which have been based on more traditional antigenic analyses using HI and MN-type assays. Only a small set of viruses from clade 3C.2a and subclade 3C.2a1 showed early signs of antigenic distancing from HK/2014. These viruses contain T135K or I192T substitutions, but it is unknown which other encoded changes, if any, contribute to over 4-fold drop in neutralization by α-HK/2014-like serum. Notably, HINT proved to be useful for directly assessing the antigenic relatedness of A(H3N2) virus isolates and viruses in human respiratory specimens, which will increase throughput and directly characterises the viruses produced in the human airway. Thus, HINT offers a valuable addition to the current laboratory tools available for analysis of antigenic relatedness.

## Materials and Methods

### Cell culture

MDCK-SIAT1 cell line [SIAT1] (IRR; Manassas, VA) were maintained by the Scientific Products and Support Branch, CDC according to the developer’s instruction. Briefly, SIAT1 cells were maintained with complete medium [Dulbecco’s modified Eagle’s medium high glucose (DMEM; Gibco) supplemented with 10% (v/v) heat-inactivated foetal bovine serum (Hyclone), 2 mM L-glutamine (Gibco), 1 mM sodium pyruvate, 100 U/ml penicillin, 100 µg/ml streptomycin (Gibco), 15 mM sodium bicarbonate] in the presence of 1 mg/ml of Geneticin (G418; ThermoFisher Scientific) at 37 °C with 5% CO_2_. Prior to experiments, cells were propagated in absence of Geneticin for at least three passages, and up to 25 passages.

### Clinical specimens and virus isolates

Influenza viruses (clinical specimens and/or virus isolates) collected during years 2012–2018 were submitted to the WHO Collaborating Centre for Surveillance, Epidemiology and Control of Influenza at the CDC for virological surveillance. A/Texas/50/2012 (H3N2) was obtained from IRR (FR-1210).

### Generation of ferret post-infection antisera

Animals were handled in compliance with guidelines of the CDC Institutional Animal Care and Use Committee (IACUC) in association with the PHS Policy, the Animal Welfare Act (USDA), and the Guide for Animal Care and Use of Laboratory Animals (National Research Council. 2011). The animal protocol was approved by the CDC IACUC committee. All procedures and manipulation of animals were conducted within a biosafety cabinet in animal biosafety level 2 (ABSL2) facilities and animal welfare was monitored daily.

Four clinical specimens containing influenza A(H3N2) viruses were selected to infect ferrets in this study: A/West Virginia/20/2012 (WV/20-3C.2), A/Hawaii/34/2015 (HI/34-3C.3b), A/Massachusetts/14/2014 (MA/14-3C.3a), and A/Alaska/140/2015 (AK/140-3C.2a). The HA1 sequences of these viruses were compared to those of the cell-propagated vaccine strains A/Victoria/361/2011 [Vic/2011] (Clade 3C), A/Texas/50/2012 [TX/2012] (clade 3C.1), A/Switzerland/9715293/2013 [SW/2013] (clade 3C.3a) and A/Hong Kong/4801/2014 [HK/2014] (clade 3C.2a) (see Fig. 1A). Infectious titre (ICP) of virus-containing human respiratory specimens was determined using HINT as described below.

Groups of four ferrets were anaesthetized by intramuscular administration of a ketamine/xylazine/atropine mixture (25 mg/kg, 2 mg/kg, and 0.05 mg/kg of body weight, respectively), and inoculated intranasally with 10^3^ ICP of virus in 1 ml preparation. Fourteen days later, ferrets were humanely euthanized, exsanguinated and sera were collected. The antisera were treated with receptor-destroying enzyme (RDE; Denka Seiken Co.) before use, by mixing 1 volume of serum with 4 volumes of RDE and incubating at 37 °C for 18–24 h. To denature the RDE, the mixture was incubated at 56 °C for 60 min followed by addition of sterile saline solution (5 vol). RDE-treated antisera were stored at −20 °C.

### Isolation and propagation of seasonal influenza A(H3N2) viruses

Virus isolation was conducted either at CDC or at the National Influenza Reference Centres (NIRCs) at CA, NY, and WI. For the purpose of the routine virological surveillance, viruses were propagated as described elsewhere^27^.

### Brief virus propagation for HINT

This protocol was used to isolate viruses from clinical specimens when preparing the reference viruses, clades 3C.2, 3C.2b, and when specified. Briefly, 50 µl of human respiratory specimen were diluted in 1 ml of virus growth medium [VGM; DMEM supplemented with 0.2% bovine serum albumin (BSA; Gibco), 25 mM HEPES (Gibco), 100 U/ml penicillin (Gibco), 100 μg/ml streptomycin (Gibco), and 3 μg/ml tosylsulfonyl phenylalanyl chloromethyl ketone treated (TPCK)-trypsin (Sigma)]. In parallel, SIAT1 cells were trypsinized and suspended to ~0.6 × 10^6^ cells/ml in VGM. The diluted clinical specimens were combined with 3 × 10^6^ SIAT1 cells in a T25 flask (Corning) and incubated at 37 °C in a 5% CO_2_ incubator for 24 hrs. Presence of virus in the cell supernatant was confirmed by measuring NA activity as described previously^37^. At 24 hrs post infection cell culture supernatants were harvested and clarified by centrifugation at 2,500 × g for 15 minutes with aliquots stored at −80 °C until use.

### High content imaging-based neutralization test

HINT consisted of 3 steps: (1) SIAT1 cell preparation, (2) virus titration to determine an appropriate virus dilution, and (3) neutralization of the virus infectivity.

*Step 1*. One day before the test, ~2 × 10^7^ SIAT1 cells were seeded onto a T162 flask and incubated at 37 °C in 5% CO_2_. The next day, cells were washed with phosphate-buffered saline (PBS) and treated with trypsin/EDTA (Life Technology) for 30 min at 37 °C. Trypsinization was stopped by the addition of complete medium and cells were centrifuged at 80 × g for 8 min at 4 °C. Cell pellet was rinsed using PBS and centrifuged again at 80 × g for 8 min at 4 °C. Finally, cells were suspended in VGM (without TPCK-trypsin) to ~6 × 10^5^ cells/ml.

*Step 2*. The test virus was serially diluted from 10^−1^ to 10^−6^ in VGM without TPCK-trypsin. The virus dilutions (100 µl), and ~3 × 10^4^ cells (50 µl; see Step 1) were transferred into a 96-well microplate (black clear-bottom plate, Costar), and incubated at 37 °C for 16–24 h, in 5% CO_2_. Supernatants were removed, cells were fixed with ice-cold methanol: acetic acid (95:5 v/v) for 1 h at −20 °C, and washed three times with PBS. Next, cells were immuno-stained with a mouse anti-NP antibody (1:1000; IRR) for 5 h at room temperature (RT°) or overnight at 4 °C, rinsed with PBS before incubating for 1 h with sheep anti-mouse IgG antibody conjugated to Alexa Fluor^TM^ 555 (1:1000; ThermoFisher Scientific) and Hoechst 33342 (4 µM; ThermoFisher Scientific). Finally, the microplates were washed three times using PBS, sealed, and scanned. Fluorescence was measured using the CellInsight^TM^ CX5 high-content imaging platform (ThermoFisher Scientific) at excitation 560 nm and emission 560 ± 25 nm. The instrument was set to scan the entire well (a total of 49 fields/well) at magnification 10x. The total number of cells (stained nuclei) and the number of infected cells (NP-positive cells) was determined (infected cell population, ICP). The virus dilution that resulted in ~1,000 infected cells per well (validated range: 150–6,000 ICP/well, see Fig. S6) was determined and used in the next step.

*Step 3*. Antiserum was 2-fold serially diluted (starting at 1:40) using VGM without TPCK-trypsin and 50 µl of each dilution were transferred into a 96-well microplate. The diluted antiserum was mixed 1:1 with diluted virus (see Step 2) and incubated for 1 h at RT. Next, 50 µl of SIAT1 cell suspension (see Step 1) were added to the virus-antiserum mixtures in the microplate, and incubated at 37 °C for 16–24 h in 5% CO_2_. The cells were fixed and stained as described in *Step 2*. Finally, the ICP in each well was determined using the CellInsight^TM^ CX5 high-content imaging platform. IC_50_ values were calculated by curve-fitting analysis using JASPR v.2 (CDC in-house software). The antiserum IC_50_ was defined as the reciprocal dilution of serum that caused 50% reduction in ICP.

### Sequencing and phylogenetic analysis

Each A(H3N2) human influenza virus was sequenced using Illumina MiSeq technology and genomes were assembled using IRMA^38^. Codon complete viral gene segments were aligned with sequences downloaded from the Global Initiative on Sharing All Influenza Data (GISAID) database following BLAST analysis to identify closely related strains. All sequences were deposited to GISAID. Phylogenetic trees of aligned gene segments were constructed using MEGA6 with neighbour-joining analysis^39^. Each analysis was run with 1,000 bootstrap iterations for statistical support. Nucleotide sequence alignments used for trees were also translated to amino acid protein sequences to compare representative viruses to A/Victoria/361/2011. AA differences among the viruses were plotted on the HA phylogenetic tree at each branch node.

### Pyrosequencing analysis

In instances when HA sequence was not available for respiratory specimens, the H3 clade determination was performed using the previously described pyrosequencing method^40^. Pyrosequencing analysis was additionally used to assess nucleotide polymorphism at triplets encoding amino acid residues 158 and 160 in the HA sequences of influenza A(H3N2) virus isolates from clades 3C.2a and 3C.2a1. The primers utilized for determining the proportion of virus population that lost the glycosylation sequon N_158_-X-T_160_ were: H3-F370, H3-R645-biotin and H3clade-R2-F445 described previously^13,40^.

### Antigenic cartography

HINT assay titration values to α-TX/2012-like, α-SW/2013-like, and α-HK/2014-like ferret sera for 2014–2017 A(H3N2) isolates were modelled with the ACMACS antigenic cartography application programming interface (API) hosted at http://www.antigenic-cartography.org/ ^5^. Individual titration values were normalized to the geometric mean of all homologous antigen-serum pair results across all test dates with a lower bound limit-of-detection of 80 for the fitted titration data. Dimensionality optimization was performed for up to 8D antigenic landscapes with the selection of 5D antigenic maps as optimal for HINT data using scree plot methodology via the *pracma* and *Deriv* libraries in R v3.4.3. Euclidean distance calculations and data frame re-structuring were performed in Python v2.7.6 with the *re*, *pandas*, and *numpy* packages. HINT data trends were linked to relevant strain metadata (*i*.*e*., clade designation and HA protein sequence) in Cloudera Hadoop database infrastructures and rendered in the Tableau v10.2 data interface engine. All Bash, SQL, R, and Python scripts utilized in the ACMACS workflow are available upon request. Computations were performed on a local virtual machine (VM) with eight 2.50 GHz Intel® Xeon® CPU E5-2670 v2 core processors.

### Protein structure visualization

The H3 HA protein structures were presented using the user-sponsored PyMOL molecular graphic system, Version 1.8.6 (Schrödinger, LLC). The AA substitutions were mapped onto the 3-dimensional structure of the A/Victoria/361/2011 (H3N2) virus HA protein (RCSB Protein Data Bank, accession number: 4WE8).

### Statistical analyses

Fold changes in HINT titres of the different AA groups and reference antigens using the nonparametric Kruskal-Wallis test with Dunns post-test. Differences between groups were considered to be significant at a P value of <0.05. Statistical analyses were performed with GraphPad Prism 5.0 (GraphPad Software, Inc., San Diego, CA).

## Disclaimer

Clinical specimens used in this study were covered under the determination of US Domestic Influenza Surveillance System. This work was intended to be public health surveillance and not research, thus it did not require human subjects’ approval or informed consent.

All experimental protocols were approved by the CDC Institutional Biosafety Committee and carried out in accordance with its guidelines and regulations. All Animal experiments were conducted in strict compliance with the guidelines of the CDC Institutional Animal Care and Use Committee (IACUC) in association with the PHS Policy, the Animal Welfare Act (U.S. Department of Agriculture [USDA]), and the Guide for Animal Care and Use of Laboratory Animals. The animal protocol was approved by the CDC IACUC.

## Supplementary information

supplementary figures

## Data Availability

All data generated or analysed during this study are included in this published article (and its Supplementary Information files).
