# Supplementary material for: Insights into the antigenic advancement of influenza A(H3N2) viruses, 2011–2018
Source: Sci Rep. 2019 Feb 25;9:2676. doi: 10.1038/s41598-019-39276-1 (PMC6389938; doi:10.1038/s41598-019-39276-1)
Supplement: Supplementary file 1 — supplementary figures [file 41598_2019_39276_MOESM1_ESM.pdf]

Insights into the antigenic advancement of influenza A(H3N2) viruses, 2011-2018

Patricia A. Jorquera [a,b,*], Vasiliy P. Mishin [a,*], Anton Chesnokov [a], Ha T. Nguyen [a,c], Brian Mann [a,c], Rebecca Garten [a], John Barnes [a], Erin Hodges [a,b], Juan De La Cruz [a,c], Xiyan Xu [a], Jackie Katz [a], David E. Wentworth [a], Larisa V. Gubareva[a#]

[a]Influenza Division, Centers for Disease Control and Prevention (CDC), Atlanta, GA, USA. 1600 Clifton Road, Atlanta GA, 30329, USA.
[b]CNI Advantage, LLC. 17 Executive Park Dr NE, Atlanta, GA 30329
[c] Battelle. Century Plaza 1, 2987 Clairmont Rd, Suite 450, Atlanta, GA 30329

*****Contributed equally
[#]Corresponding author

Correspondence to: lqg3@cdc.gov
Mail Stop G-16, 1600 Clifton Road, Atlanta GA, 30329, USA.

Figure S1

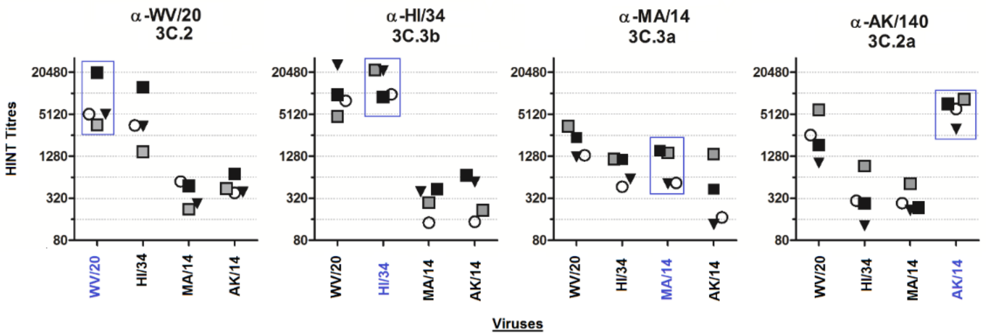

Ferret antiserum	clade	ferret	Viruses, HINT titres (fold)			
			A/WV/20/2012	*A/HI/34/2015*	*A/MA/14/2014*	*A/AK/140/2015*
α-WV/20	3C.2	1	3612 (1)	1483 (2)	222 (16)	438 (8)
		2	5123 (1)	3430 (1)	269 (19)	393 (13)
		3	5146 (1)	3549 (1)	557 (9)	384 (13)
		**4***	**20165 (1)**	12430 (2)	479 (42)	713 (28)
		Geometric Mean	6620 (1)	3870 (1.7-fold)	355 (19-fold)	467 (14-fold)
α-HI/34	3C.3b	**5***	25833 (1)	21682 (1)	397 (55)	547 (40)
		6	9751 (1)	9047 (1)	433 (21)	683 (13)
		7	4780 (5)	22045 (1)	278 (79)	214 (103)
		8	8007 (1)	9850 (1)	143 (69)	147 (67)
		Geometric Mean	9909 (1.5-fold)	14366 (1)	288 (50-fold)	329 (44-fold)
α-MA/14	3C.3a	**9***	2388 (1)	1168 (1)	1550 (1)	434 (4)
		10	1252 (0.4)	603 (1)	516 (1)	135 (4)
		11	3463 (0.4)	1167 (1)	1440 (1)	1366 (1)
		12	1320 (0.4)	469 (1)	531 (1)	170 (3)
		Geometric Mean	1923 (0.5-fold)	788 (1.1-fold)	884 (1)	341 (2.6-fold)
α-AK/140	3C.2a	**13***	1851 (4)	273 (26)	236 (31)	7224 (1)
		14	2579 (2)	295 (21)	273 (22)	6098 (1)
		15	5929 (1)	926 (9)	520 (16)	8423 (1)
		16	1022 (3)	129 (24)	214 (15)	3123 (1)
		Geometric Mean	2319 (2.5-fold)	313 (19-fold)	291 (20-fold)	5834 (1)

Sup. Figure 1: HINT titres for ferret antisera collected at day 14 post infection with human respiratory specimens. Homologous response is shown in blue. Individual values for each ferret serum is shown below the graphs. Antisera chosen for further analysis are indicated with an asterisk (ferrets 4, 5, 9 and 13).

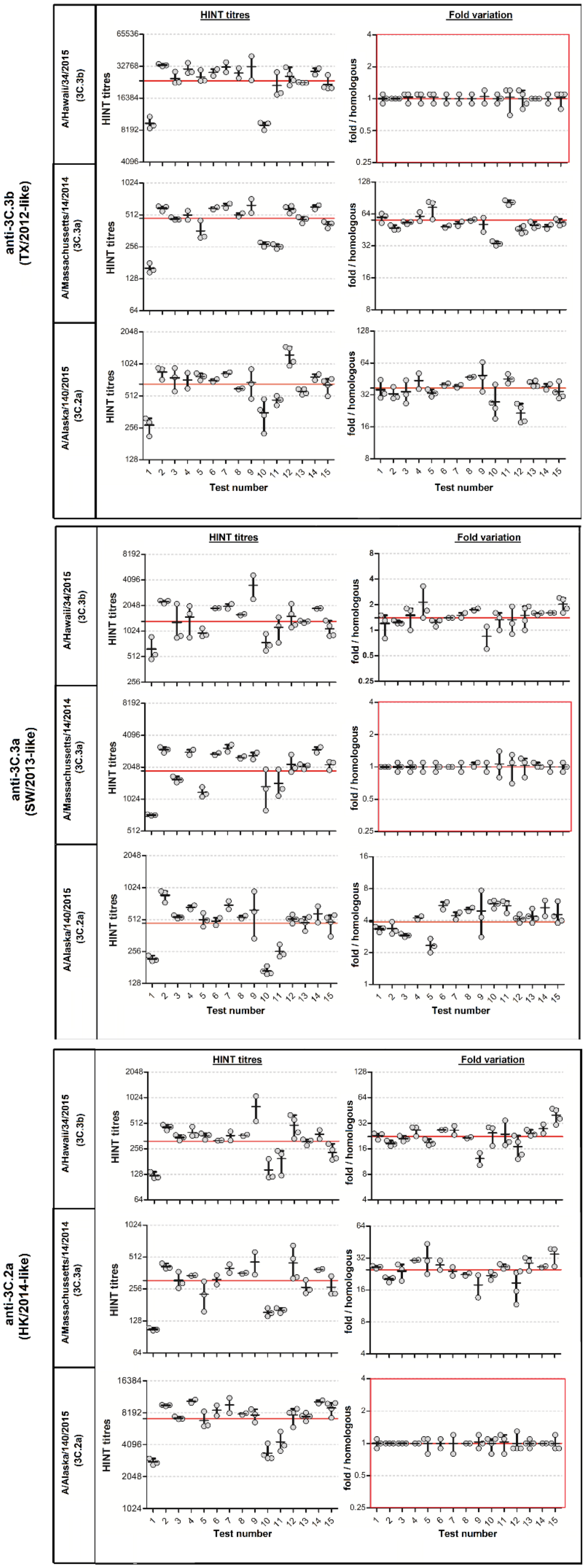

Figure S2

Sup.Fig.2: HINT reproducibility. Reference viruses and ferret antisera were tested in 15 independent tests. Data are presented as HINT neutralization titres and fold variation in HINT titre compared to homologous geometric mean titre. The horizontal red line indicates the geometric mean value for the entire population. Plots with a red frame indicate homologous pairs.

Figure S3

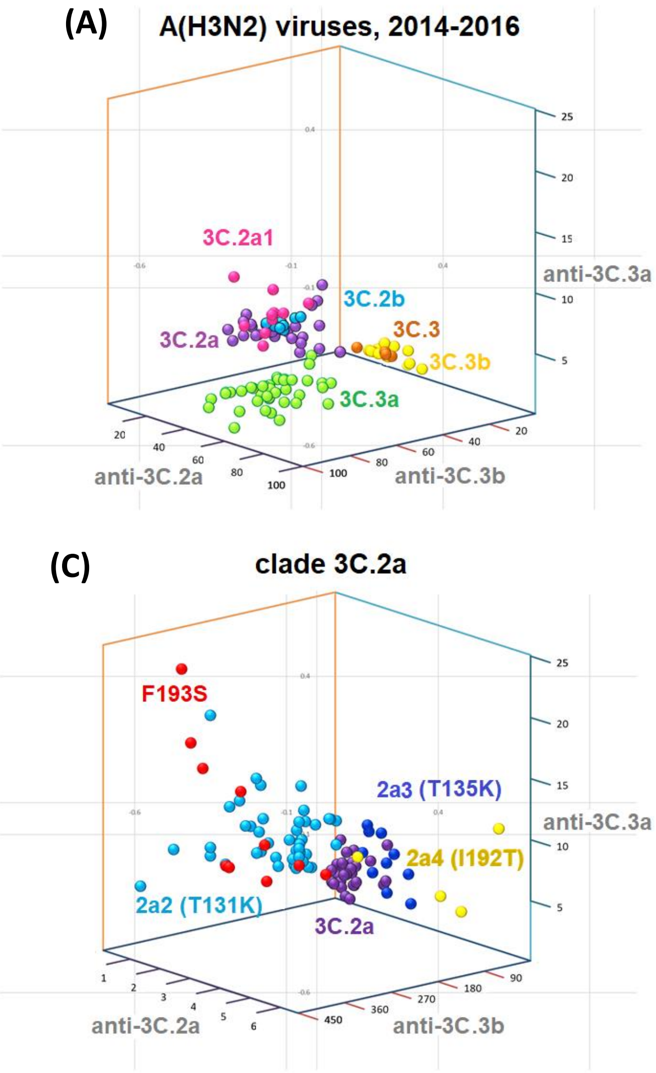

Sup. Fig. 3. Neutralization profile of influenza A(H3N2) viruses tested with three reference antisera. Individual groups are color-coded for an easier visualization. **(A)** Earlier Influenza A(H3N2) viruses circulating during Jan/2014-Dec/2016. **(B)** clade 3C.3a viruses isolated in Oct/2014-Jun/2017 ;**(C)** clade 3C.2a isolated in Nov/2014-Nov/2017, and **(D)** clade 3C.2a1 isolated in Sept/2015- Dec/2017. Axes indicate the fold reduction in HINT titres by the clade-specific antisera.

Figure S4

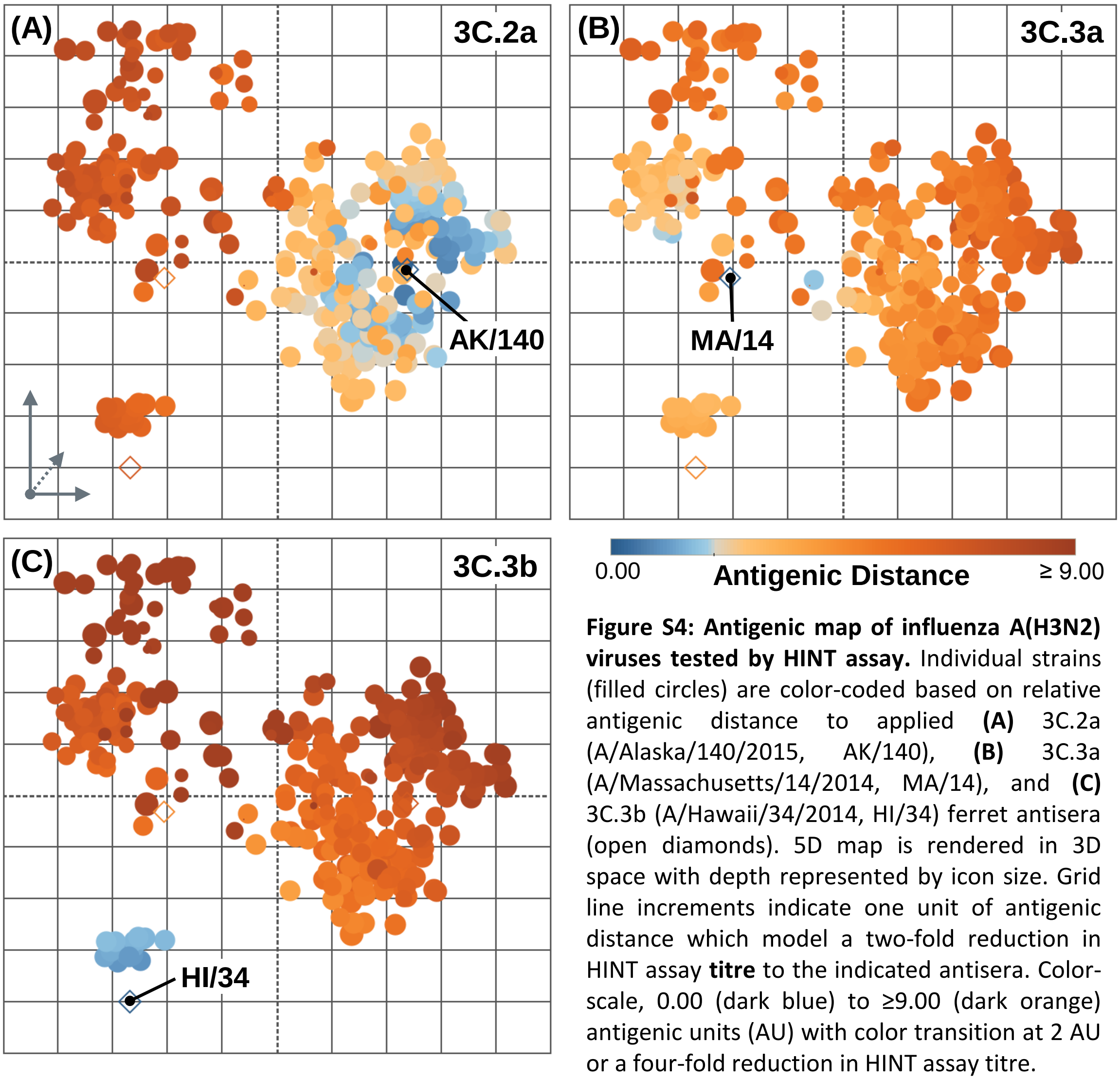

Figure S4: Antigenic map of influenza A(H3N2) viruses tested by HINT assay. Individual strains (filled circles) are color-coded based on relative antigenic distance to applied **(A)** 3C.2a (A/Alaska/140/2015, AK/140), **(B)** 3C.3a (A/Massachusetts/14/2014, MA/14), and **(C)** 3C.3b (A/Hawaii/34/2014, HI/34) ferret antisera (open diamonds). 5D map is rendered in 3D space with depth represented by icon size. Grid line increments indicate one unit of antigenic distance which model a two-fold reduction in HINT assay **titre** to the indicated antisera. Color-scale, 0.00 (dark blue) to ≥9.00 (dark orange) antigenic units (AU) with color transition at 2 AU or a four-fold reduction in HINT assay titre.

Figure S5

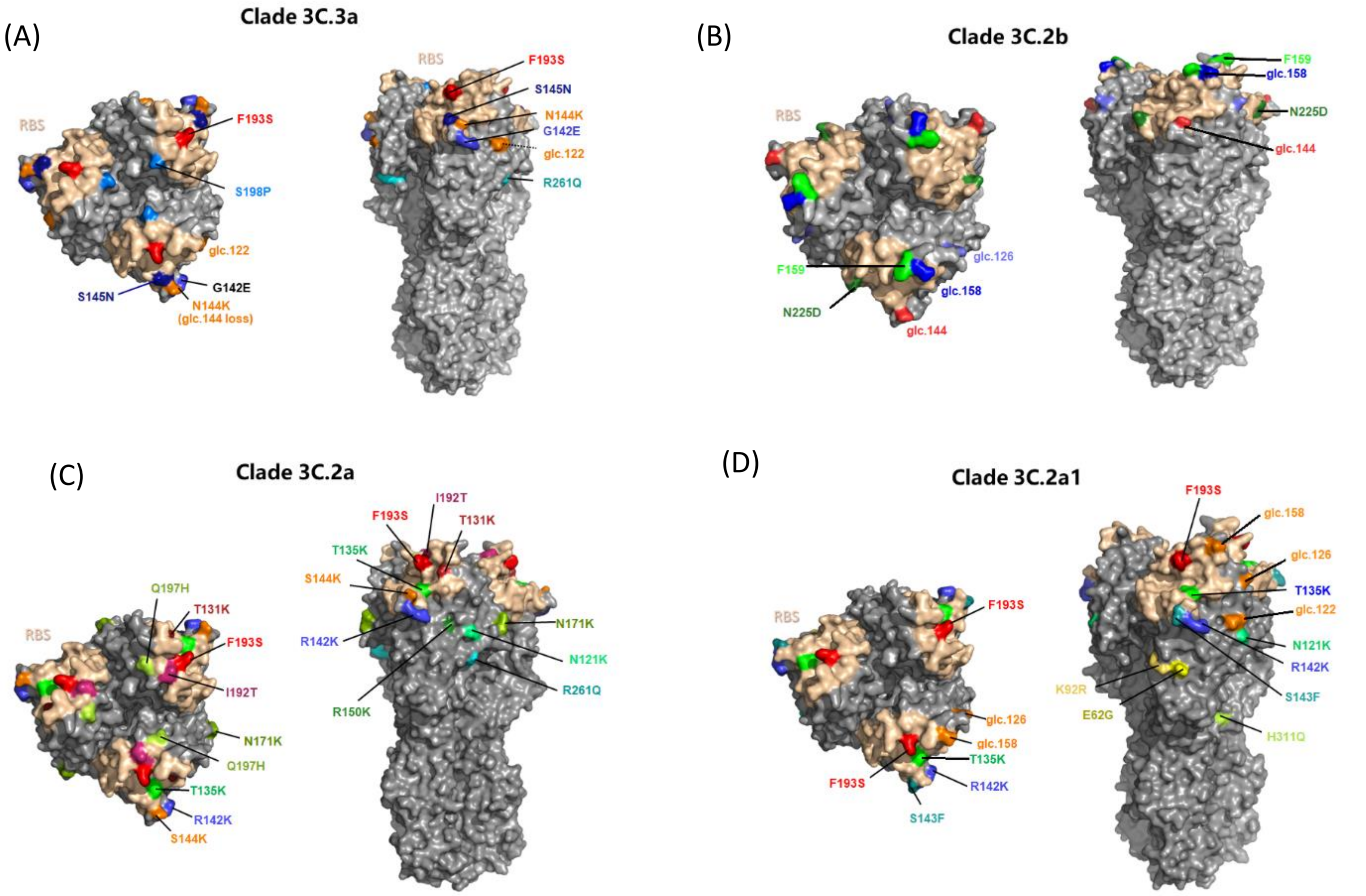

Fig. S5: 3D visualization of the HA trimer highlighting HA1 AA changes detected in A(H3N2) viruses. The H3 HA protein structures were presented using the user-sponsored PyMOL molecular graphic system, Version 1.8.6 (Schrödinger, LLC). The individual AA substitutions seen in clade A) 3C.3a, B)3C.2b, C)3C.2a and D)3C.2a1 were mapped onto the 3-dimensional structure of the A/Victoria/361/2011 (H3N2) virus HA protein (RCSB Protein Data Bank, accession number: 4WE8).

Figure S6

(A)

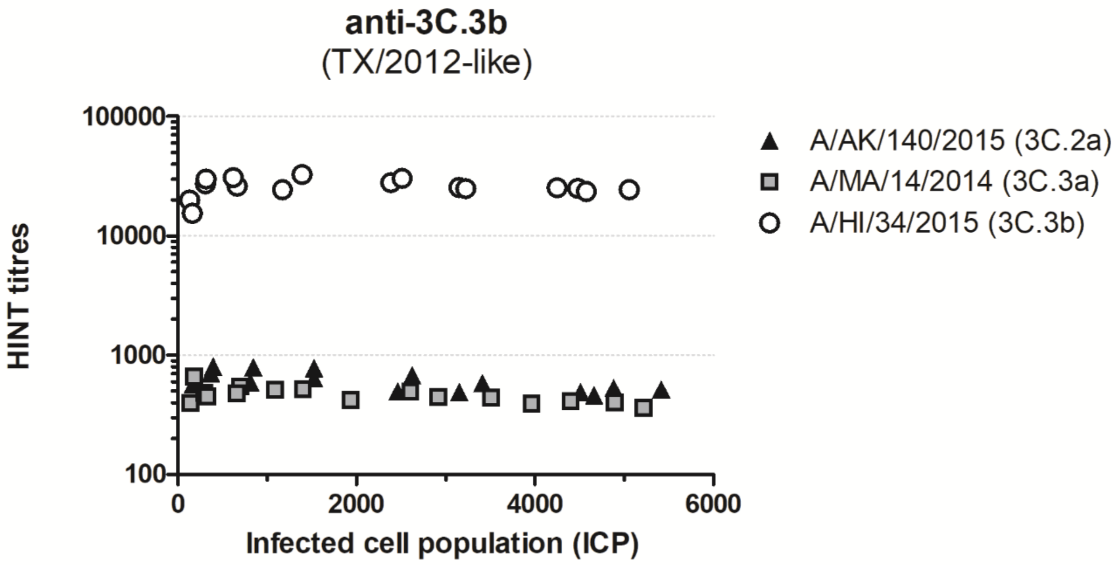

(B)

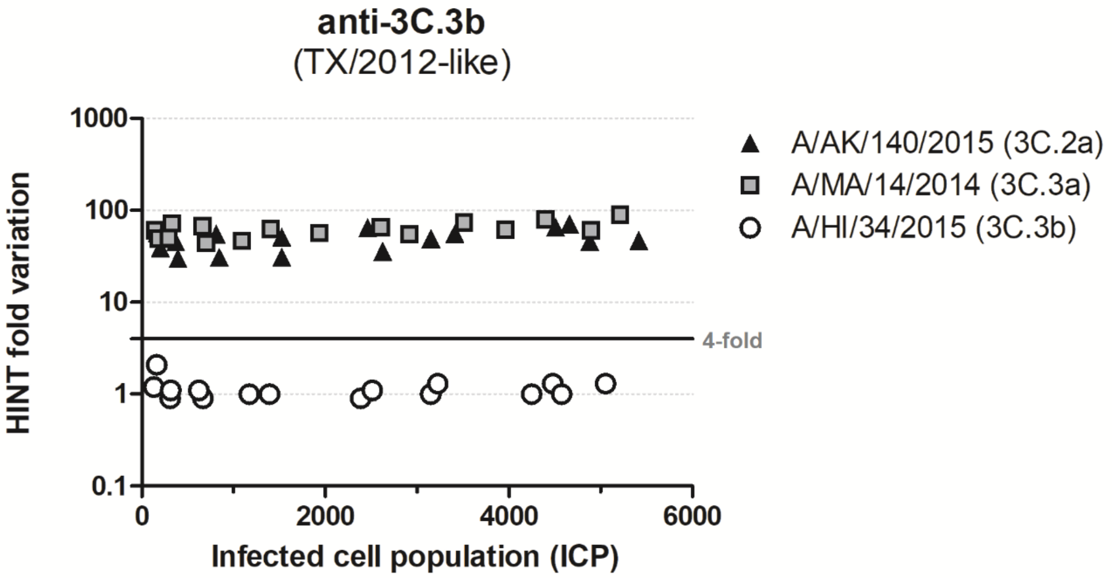

Sup. Fig.6: Effect of ICP on neutralization by anti-3C.3b reference antiserum. Increasing amounts of reference viruses (100 to 6000 ICP/well) were tested in HINT using the anti-3C.3b reference antiserum as source of neutralizing antibodies. Data is presented as HINT titres (A) or HINT fold variation (B) versus infectious dose. HINT fold variation were calculated based to 1000 ICP of A/HI/34/2015

The ability of anti-3C.3b serum to neutralize the reference viruses was not affected by the inoculation dose, showing linear inhibition between 100 to 6000 ICP.
